# Cognitive limits of larval *Drosophila*: testing for conditioned inhibition, sensory preconditioning, and second-order conditioning

**DOI:** 10.1101/lm.053726.122

**Published:** 2024-05

**Authors:** Edanur Sen, Amira El-Keredy, Nina Jacob, Nino Mancini, Gülüm Asnaz, Annekathrin Widmann, Bertram Gerber, Juliane Thoener

**Affiliations:** 1 Department of Genetics, Leibniz Institute for Neurobiology, 39118 Magdeburg, Germany; 2 Department of Genetics, Faculty of Agriculture, Tanta University, 31111 Tanta, Egypt; 3 Department of Molecular Neurobiology of Behavior, University of Göttingen, 37077 Göttingen, Germany; 4 Otto von Guericke University Magdeburg, Institute of Biology, 39106 Magdeburg, Germany; 5 Center for Behavioral Brain Sciences, 39106 Magdeburg, Germany

## Abstract

*Drosophila* larvae are an established model system for studying the mechanisms of innate and simple forms of learned behavior. They have about 10 times fewer neurons than adult flies, and it was the low total number of their neurons that allowed for an electron microscopic reconstruction of their brain at synaptic resolution. Regarding the mushroom body, a central brain structure for many forms of associative learning in insects, it turned out that more than half of the classes of synaptic connection had previously escaped attention. Understanding the function of these circuit motifs, subsequently confirmed in adult flies, is an important current research topic. In this context, we test larval *Drosophila* for their cognitive abilities in three tasks that are characteristically more complex than those previously studied. Our data provide evidence for (i) conditioned inhibition, as has previously been reported for adult flies and honeybees. Unlike what is described for adult flies and honeybees, however, our data do not provide evidence for (ii) sensory preconditioning or (iii) second-order conditioning in *Drosophila* larvae. We discuss the methodological features of our experiments as well as four specific aspects of the organization of the larval brain that may explain why these two forms of learning are observed in adult flies and honeybees, but not in larval *Drosophila*.

Investigations of *Drosophila melanogaster* have led to the discovery of evolutionarily conserved mechanisms for associative learning and memory (Dudai et al. 1976; Heisenberg et al. 1985; Tully and Quinn 1985). The application of convenient methods for cell-specific transgene expression (Brand and Perrimon 1993; Pfeiffer et al. 2010) then enabled follow-up analyses to reveal the neuronal circuits underlying this simple form of cognition (Zars et al. 2000; Guven-Ozkan and Davis 2014; Gerber and Aso 2017; Cognigni et al. 2018; Boto et al. 2020).

The potential of larval *Drosophila* for learning and memory research was realized early on (Aceves-Piña and Quinn 1979), and with renewed interest when paradigms were established for the association of odors and taste reward (Scherer et al. 2003; Neuser et al. 2005) and punishment (Gerber and Hendel 2006; Niewalda et al. 2008; El-Keredy et al. 2012), and between odors and the optogenetic activation of brain reward neurons (Schroll et al. 2006). Larvae possess about 10 times fewer neurons than adult flies, but feature adult-like circuit motifs—for example, in the olfactory pathways (Gerber and Stocker 2007; Vosshall and Stocker 2007; Diegelmann et al. 2013; Thum and Gerber 2019; Eschbach and Zlatic 2020). The larva's nervous system has been partially mapped into a light-microscopic cell atlas (Li et al. 2014), and transgenic driver strains can be generated to manipulate neurons of interest in the context of learning experiments (Rohwedder et al. 2016; Saumweber et al. 2018). Furthermore, the electron microscopic reconstruction of the chemical-synapse connectome of a first-instar larval brain has revealed unexpected circuit complexity in the mushroom body (Eichler et al. 2017; Winding et al. 2023), a central-brain structure that is essential for a number of mnemonic processes in insects, including for associative memory between odors and taste reward (for reviews, see Menzel and Benjamin 2013). The analysis and interpretation of these circuit motifs, subsequently confirmed in adult flies (Takemura et al. 2017; Zheng et al. 2018; Li et al. 2020), now largely define the research agenda of the field.

In this context, we probe the cognitive limits associated with a mushroom body that is numerically as simplified as in larval *Drosophila*. We test these animals in three tasks, well established in experimental psychology (Rescorla 1988a,b), that are characteristically more complex than the paradigms hitherto applied in larvae: conditioned inhibition, sensory preconditioning, and second-order conditioning.

## Conditioned inhibition

In a typical associative learning task, a cue (A) is presented together with a reward (+) (in Pavlovian terminology these would be referred to as the conditioned and unconditioned stimulus). Such paired training (A+) is said to establish “conditioned excitation” because the cue *excites* the expectation of the reward to occur and prompts behavior *in anticipation of receiving* it. In contrast, “conditioned inhibition” refers to the opposing process, which allows the cue to *inhibit* the expectation of the reward to occur, prompting behavior *in anticipation of not receiving* it (Rescorla 1969). Conditioned inhibition can be established by training the subjects such that whenever the reward is presented the cue is not, and vice versa (unpaired training: +/A). In other words, paired training establishes A as a predictor of reward occurrence (conditioned excitation), whereas unpaired training establishes A as a predictor for the reward's nonoccurrence (conditioned inhibition). Learning through unpaired training is characteristically complex, because although it is about the reward, it takes place at a moment when the reward is not physically present.

Opposing effects of paired versus unpaired training have been reported in larval *Drosophila* (Saumweber et al. 2011; Schleyer et al. 2018, 2020), adult flies, and honeybees *Apis mellifera* (Bitterman et al. 1983; Matsumoto et al. 2012; Barth et al. 2014; Jacob and Waddell 2020). Here, we confirm and extend these results in larval *Drosophila* with respect to hallmark features of conditioned inhibition, using presentations of a sugar taste reward unpaired from an odor cue.

## Sensory preconditioning

Sensory preconditioning (Brogden 1939) refers to the learning that results from two cues A and B—say the visual appearance and the song of a bird—occurring together, thus establishing their combination into a psychological object. This allows for pattern completion if one of the cues is not physically present—for example, when the song of the wood pewee calls up its visual appearance. Sensory preconditioning can be demonstrated in a two-stage experiment. In phase (i), AB are presented, and then in phase (ii), A+ is trained, followed by a test of B. Responding to B during the test would be indicative of sensory preconditioning. The characteristic complexity of such sensory preconditioning is evident first in that it takes place in the absence of reinforcement in phase (i). It is evident second in that it requires chained processing during phase (ii) such that by virtue of the previously established AB association cue A calls up B, which is then associated with +, and/or chained processing during the test such that, again by virtue of the previously established AB association, cue B calls up A, which then calls up the A+ association (Molet et al. 2012). Either way, sensory preconditioning hinges on the AB association established in phase (i).

Sensory preconditioning has been shown in adult flies and bees (Müller et al. 2000; Brembs and Heisenberg 2001; Martinez-Cervantes et al. 2022), including for binary odor compounds as cues A and B. We sought to establish sensory preconditioning in larvae, likewise between the elements of binary odor compounds.

## Second-order conditioning

Second-order conditioning (Pavlov 1927; Rescorla 1980) refers to the observation that when a cue A is firmly associated with a reward it can itself have a rewarding effect—even when the reward is not physically present. For example, once humans have learned that money can buy chocolate, money can itself act as a reward of the second order. Experimentally, second-order conditioning can be demonstrated by first (i) training A+, and then (ii) presenting AB, followed by a test of B revealing a response. For cues repeatedly experienced in succession before a reward, for example, O-then-X-then-A and only then the reward, second-order conditioning allows the staggered formation of associations for cues such as O, which would be temporally too far removed from the reward if they were trained in isolation. In other words, second-order conditioning can identify the earliest reward-predicting cue, allowing temporally distant goals to be pursued in a chained manner.

Second-order conditioning has been observed in adult flies and bees (Takeda 1961; Bitterman et al. 1983; Brembs and Heisenberg 2001; Hussaini et al. 2007; Tabone and De Belle 2011; Yamada et al. 2023), again including for binary odor compounds as cues. We sought to establish second-order conditioning for larvae, using binary odor compounds as well.

## Materials and Methods

### Animals, materials, and chemicals

*Drosophila melanogaster* larvae were raised in mass culture on standard cornmeal–molasses food and maintained at 25°C, 60%–70% relative humidity, and a 12 h:12 h light–dark cycle. For behavioral experiments, 5-d-old, third-instar, feeding-stage, wild-type Canton Special larvae of either prospective sex were used. Cohorts of approximately 30 larvae were collected from the food vials, rinsed in water, collected in a water droplet, and then used for experiments.

For behavioral experiments, Petri dishes of 9 cm diameter (Nr. 82.1472 Sarstedt) were filled with 1% agarose solution as the substrate (PUR; electrophoresis grade; CAS: 9012-36-6, Roth) or with fructose as the sugar taste reward (+) added to the agarose solution (2 M; purity 99%; CAS: 57-48-7 Roth). For odor presentation, either custom-made Teflon containers or Petri dish lids equipped with filter papers were used. The Teflon containers were of 5 mm diameter with perforated lids with five to 10 holes, each of ∼0.5 mm diameter. These were filled with 10 µL of odor solution before the experiment and used for 1 d. The aforementioned Petri dish lids were equipped with four filter papers; each of these filter papers was loaded with 5 µL of the respective odor solution shortly before each experiment. When two odors were presented in the compound, 5 µL of each odor solution was used per filter paper; these were renewed after each experiment.

As the odor substances, either *n-*amylacetate (AM; CAS: 628-63-7, Merck; diluted 1:20 in paraffin oil; CAS: 8042-47-5, AppliChem) or 1-octanol was used (1-OCT; CAS: 111-87-5; Merck; undiluted). Paraffin is without behavioral significance in larval *Drosophila* (Saumweber et al. 2011).

### Behavioral experiments

#### Effects of PAIRED versus UNPAIRED training with one training trial

**Figure 1.**
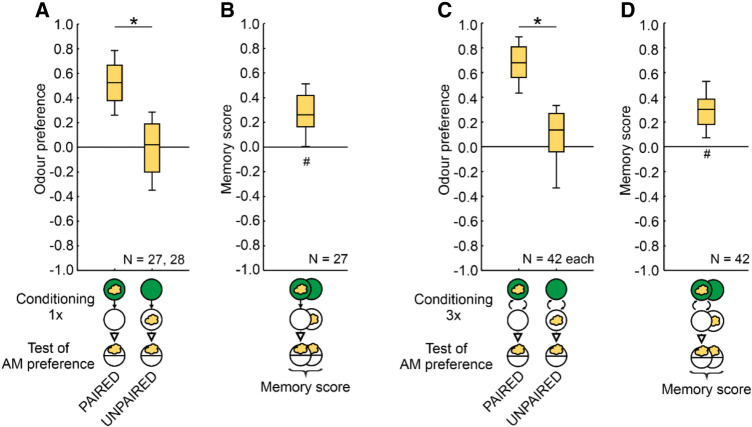
Odor–sugar associative memory in larval *Drosophila*. (*A*–*D*) *Drosophila melanogaster* larvae received either PAIRED training of an odor (yellow cloud, *n*-amylacetate) and a fructose sugar reward presented on the same Petri dish (green circle), or they received the sugar reward separately (UNPAIRED training). To equate for training duration and handling, blank periods (open circle) were added for the PAIRED case. The sequence of events during training was as depicted in half of the cases and the reverse in the other half of the cases (not shown). After training, the larvae were tested for their odor preference (Equation 1), and a memory score was calculated from the difference between PAIRED and UNPAIRED training (Equation 2). (*A*) After one-trial PAIRED training the larvae show higher odor preferences than after UNPAIRED training. (*B*) The resulting memory scores indicate appetitive associative memory. (*C*,*D*) Corresponding results are observed after three training trials. Box plots show the median as the midline, the 25%/75% quantiles as box boundaries, and 10%/90% quantiles as whiskers. Sample sizes are indicated within the figure. (#) Significance from zero in an OSS test, (*) significance in an MWU test. Statistical results and source data are in Supplemental Table S1.

Experiments followed a standard, one-odor, single-training-trial protocol (Fig. 1A; Saumweber et al. 2011; Weiglein et al. 2019; for a manual, see Michels et al. 2017). Larvae underwent either paired or unpaired presentations of AM as the odor and the sugar taste reward (+), followed by a preference test for the odor.

For paired training (PAIRED), two containers with AM were located on opposite sides of a Petri dish filled with sugar-supplemented agarose. Cohorts of ∼30 larvae were placed in the middle of the Petri dish and left undisturbed for 2.5 min to disperse through the Petri dish (AM+). Subsequently, the larvae were collected with a brush and transferred to a second “blank” Petri dish containing neither the sugar reward nor the odor and left free to move about the Petri dish for a further 2.5 min; in this case, two empty odor containers (EM) were placed on the Petri dish. In half of the cases, this sequence was as mentioned (AM+/EM), whereas in the other half of the cases, it was the reverse (EM/AM+). Subsequently, the larvae were transferred to a test Petri dish with agarose but no sugar added (unless mentioned otherwise); in this case, an odor container with AM was placed on one side, and an EM container was placed on the opposite side of the Petri dish. After 3 min, the number of larvae on each side (@AM-side and @EM-side, respectively) and the typically <12% of larvae on a 10-mm-wide middle zone were counted, including the larvae crawling up the sidewalls of the Petri dish; only larvae crawling up the lid of the Petri dish were excluded (<5%). From these numbers, a preference score for the odor was calculated as

(1) $$\mathrm{Odour}\;\mathrm{preference}=\frac{@\mathrm{AM}\;-\;@\mathrm{EM}}{\mathrm{Total}}.$$

Thus, odor preference scores may range from +1 to −1, with positive values indicating an approach to the odor and negative values indicating avoidance.

For unpaired training (UNPAIRED), an independent cohort of larvae was likewise placed in Petri dishes, in this case, however, featuring either only agarose with the sugar reward added but no odor (EM+) or the odor but no sugar reward added to the agarose (AM). Again, either the aforementioned sequence (EM+/AM) or the reverse sequence (AM/EM+) was used, followed by the test for odor preference as described above.

To quantify associative memory, a memory score was calculated based on the odor preference scores after PAIRED and UNPAIRED training as

(2) $$\begin{array}{rl} & \mathrm{Memory}\;\mathrm{score}=\frac{\mathrm{PAIRED}\;-\mathrm{UNPAIRED}\;}{2}. \end{array}$$

Values for the memory score may thus range from +1 to −1, with positive values indicating appetitive associative memory, whereas negative values would indicate aversive associative memory.

To quantify the effect of using different sequences of odor and sugar reward presentation, we separately calculated a sequence index for the odor preference scores after PAIRED and UNPAIRED training as follows:

(3) $$\mathrm{Sequence}\;{\mathrm{index}}_{\mathrm{PAIRED}}=\frac{(\mathrm{EM}/\mathrm{AM}+)-\;(\mathrm{AM}+/\mathrm{EM})}{2},$$

(4) $$\mathrm{Sequence}\;{\mathrm{index}}_{\mathrm{UNPAIRED}}=\frac{\left(\mathrm{EM}+/\mathrm{AM})-(\mathrm{AM}/\mathrm{EM}+\right)}{2}.$$

Values of the sequence index may thus range from +1 to −1. Positive values indicate higher odor preferences when the odor came last during training (EM/AM+ or EM+/AM) as compared to when the odor came first (AM+/EM or AM/EM+). Conversely, negative sequence indices indicate higher preferences when the odor came first (AM+/EM or AM/EM+) as compared to when the odor came last (EM/AM+ or EM+/AM). The arrangement of the numerator and thus the signs of the sequence index are arbitrary in a technical sense. The way we arranged them ensures that, according to the model that we want to test, conditioned inhibition should lead to negative sequence indices for the UNPAIRED groups (for more details on the underlying rationale, see Results section). We then arranged the numerator for the PAIRED groups such that for the data in Figure 4D,H any trend in the sequence indices of the PAIRED group would have the same sign as for the UNPAIRED groups; this is conservative because it underestimates the difference between the PAIRED and the UNPAIRED groups.

#### Effects of PAIRED versus UNPAIRED training with three training trials

All procedures were the same as in the preceding section, except that two additional training trials were performed in immediate succession.

#### Sensory preconditioning

To test for sensory preconditioning, larvae first underwent a preconditioning phase exposing them to two odors, followed by an odor–reward conditioning phase for one of these odors and a test of preference for the respective other odor (see Fig. 5).

During preconditioning, the larvae received the two odors AM and 1-OCT either presented together as a COMPOUND or temporally SEPARATED from each other, in both cases on Petri dishes with only pure agarose; to equate for the total duration of the experiment and for handling, we added a blank trial for the COMPOUND case. The sequence of trial types during preconditioning was balanced across repetitions of the experiment. Three trials of preconditioning were followed by a conditioning phase, in which the larvae in the experimental groups received a single trial of PAIRED presentation of AM and the sugar reward (AM+/EM or EM/AM+). The larvae in the control groups received neither the odor nor the sugar reward (HANDLING), only the SUGAR (EM+/EM or EM/EM+), or only the ODOR (AM/EM or EM/AM). In the following test, the larvae were assayed for their preference for 1-OCT, determined with due adjustment according to Equation 1.

To quantify the impact of preconditioning as the difference in odor preference between the SEPARATED and the COMPOUND cases, a difference index was calculated as

(5) $$\begin{array}{rl} & \mathrm{Difference}\;\mathrm{index}=\frac{\mathrm{COMPOUND}-\mathrm{SEPARATED}\;}{2}. \end{array}$$

Accordingly, positive values indicate a higher odor preference in larvae that received the odors in COMPOUND during preconditioning as compared to larvae that received them SEPARATED; negative values indicate the opposite. Sensory preconditioning as an associative phenomenon would be indicated by higher difference indices in the experimental groups than in the control groups.

For odor presentation, the Petri dish lids were equipped with four (during preconditioning and conditioning) or two (during the test) filter papers. One minute before the lid was placed on the Petri dish, the filter papers were loaded with the respective odors.

Variations on the paradigm are mentioned within the Results section.

#### Second*-*order conditioning

To test for second-order conditioning, the larvae first underwent a conditioning phase to establish an odor–reward association (first-order conditioning). This was followed by a second-order conditioning phase in which the previously rewarded odor was presented together with a novel odor. Then the larvae were tested for their preference for this novel “target” odor (see Fig. 7).

During first-order conditioning, the larvae in the experimental groups received a single PAIRED presentation of AM and the sugar reward (AM+/EM or EM/AM+). The larvae in the control groups received neither the odor nor the sugar reward (HANDLING), only the SUGAR (EM+/EM or EM/EM+) or only the ODOR (AM/EM or EM/AM). This was followed by the second-order conditioning phase, during which the previously rewarded odor AM was presented either in COMPOUND with a novel, target odor (1-OCT) or temporally SEPARATED from the target odor; in both cases, Petri dishes with only pure agarose were used. To equate for the total duration of the experiment and for handling, we added a blank trial for the COMPOUND case. The sequence of trial types during second-order conditioning was balanced across repetitions of the experiment, too. In the following test, the larvae were assayed for their preference for 1-OCT, determined with due adjustment according to Equation 1.

To quantify the impact of the second-order conditioning phase, the difference between the SEPARATED and the COMPOUND cases was determined by calculating a difference index according to Equation 5. Positive values therefore indicate a higher odor preference in larvae that received the odors in COMPOUND during second-order conditioning as compared to larvae that received them SEPARATED; negative values indicate the opposite. Second-order conditioning as an associative phenomenon would be indicated by higher difference indices in the experimental groups than in the controls.

Variations on the paradigm are mentioned within the Results section.

### Statistics

Nonparametric statistics were performed throughout (Statistica 13, RRID:SCR_014213, StatSoft Inc.). To test whether values are significant relative to chance level (zero), one-sample sign (OSS) tests were used. To compare across multiple independent groups, Kruskal–Wallis (KW) tests with subsequent pairwise comparisons by Mann–Whitney *U* (MWU) tests were performed. To ensure a within-experiment error rate of <5%, Bonferroni–Holm corrections (Holm 1979) were applied. Data are shown as box plots with the median as the middle line, the 25% and 75% quantiles as box boundaries, and the 10% and 90% quantiles as whiskers.

The results of the statistical tests and the source data of all experiments are documented in the data file Supplemental Table S1.

## Results

### PAIRED and UNPAIRED training modulate odor preferences in an opposing manner

Odor preferences after one trial of PAIRED odor–reward training are higher than after presentations of odor temporally UNPAIRED from the reward (Fig. 1A). Such a difference indicates appetitive associative memory and is reflected in a positive memory score (Fig. 1B). Corresponding results were obtained after three training trials (Fig. 1C,D). However, such results do not allow one to conclude whether learning has taken place through PAIRED training, through UNPAIRED training, or both. Through PAIRED training the larvae may learn that the odor predicts the occurrence of the reward, leading to an increase in odor preference. Through UNPAIRED training they may learn the opposite—namely, that the odor predicts the nonoccurrence of the reward, leading to a decrease in odor preference. This begs the question as to what the baseline level of odor preference is, cleared of the associative effects of the training experience.

**Figure 2.**
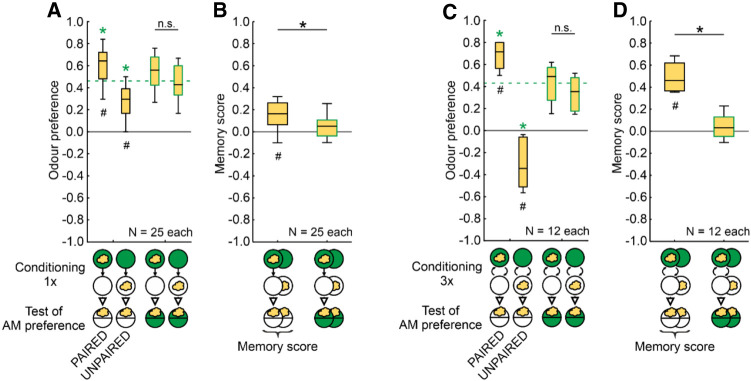
PAIRED and UNPAIRED training modulates odor preferences in an opposing manner. (*A*–*D*) Larvae were trained with PAIRED or UNPAIRED presentations of odor and the sugar reward and tested for their odor preference, either in the absence or in the presence of the reward. (*A*) After one-trial PAIRED training and when tested in the absence of the reward, the larvae show higher odor preferences than after UNPAIRED training (two box plots to the *left*). However, when the larvae were tested in the presence of the reward, this difference was abolished (two box plots to the *right*); their respective odor preference values can thus be pooled to represent baseline odor preference, cleared of the influence of associative memories. The green stippled line represents the median of these pooled preference values. (*B*) The corresponding memory scores are higher when the animals were tested in the absence than in the presence of the reward (innate odor preferences are unaffected by the presence of the reward: Supplemental Fig. S1). (*C*,*D*) The same pattern of results is observed after three training trials. Note that after UNPAIRED training and when the test is carried out in the absence of the reward, larvae show avoidance of the odor (*C*, second from *left*). (#) Significance from zero in an OSS test, (*) significance in an MWU test, (n.s.) nonsignificance in an MWU test. A green * symbol refers to significance in an MWU test against baseline (i.e., against the pooled preferences when the animals were tested in the presence of the reward). Statistical results and source data are in Supplemental Table S1. Other details as in Figure 1.

In larval *Drosophila* such a baseline odor preference can be determined by testing the animals in the presence of the reward. That is, the difference in odor preference after PAIRED versus UNPAIRED training is abolished when the testing is carried out in the presence of the reward (Fig. 2A), leading to memory scores indistinguishable from chance level (Fig. 2B) (“innate” odor preferences in experimentally naive animals are not altered by the presence of the reward: Supplemental Fig. S1). These findings can be grasped by the notion of appetitive associative memory supporting a learned search for the reward, which is abolished when the sought-for reward is present (Gerber and Hendel 2006; Schleyer et al. 2011). For the current context, the important point is that the residual odor preference that is observed when the reward is present during testing reflects the odor preference specifically cleared of the influence of associative memories. These pooled preferences, represented by their median as the stippled line in Figure 2A, can, therefore, be used as a baseline against which the associative effects of PAIRED and UNPAIRED training can be measured. This shows that odor preferences after PAIRED training are increased relative to baseline, whereas after UNPAIRED training they are decreased (Fig. 2A). The same is observed after three training trials (Fig. 2C,D).

We conclude that PAIRED versus UNPAIRED training have opposite effects on odor preference, confirming earlier reports (Saumweber et al. 2011; Schleyer et al. 2011, 2015a,b, 2018, 2020; Paisios et al. 2017; Weiglein et al. 2019). From the present study and these earlier reports, however, it remains unresolved whether the opposing effects of PAIRED versus UNPAIRED training indeed reflect conditioned excitation versus inhibition. Why is that?

### Does UNPAIRED training establish conditioned inhibition or learned inattention?

**Figure 3.**
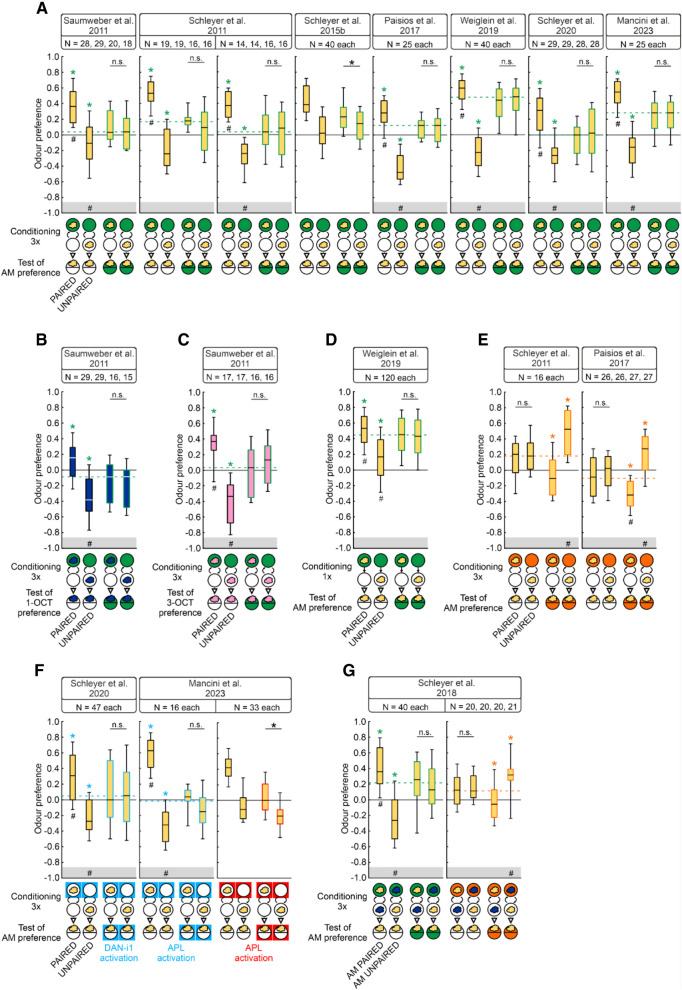
UNPAIRED training with reward can establish odor avoidance. (*A*–*G*) Survey of previously published experiments that revealed both PAIRED and UNPAIRED memory against baseline odor preferences (stippled lines) using different odors (clouds) (yellow, *n*-amylacetate; blue, 1-octanol; pink, 3-octanol) and rewards (circles/squares) (fructose, green; optogenetic activation of the indicated neurons, blue/red for ChR2-XXL/Chrimson), or quinine as a punishment (orange). As indicated, the training involved either one-odor “absolute” conditioning (*A*–*F*), or two-odor differential conditioning followed by single-odor testing (*G*), with the indicated number of training trials. Considering the total of 15 reward cases, in 11 out of the 13 data sets in which the baseline could be determined, UNPAIRED training established odor preferences that were both below the baseline and reflected odor avoidance (85%). This argues against learned inattention as the psychological mechanism for UNPAIRED memory. In two of the cases, the baseline could not be determined because there were significant differences between the PAIRED and UNPAIRED trained groups tested in the presence of the reinforcer; this is indicated by the absence of the gray horizontal boxes at the *bottom* of the panels. For the special case of one-trial reward training, see the discussion in the body text and Figure 4C. The three cases of punishment reveal that UNPAIRED training established increased odor attraction, likewise arguing against learned inattention. Critical evidence for conditioned inhibition is highlighted by placing the # symbol in the gray box at the *bottom* of the panels. (#) Significance from zero in an OSS test, (*) significance in an MWU test, (n.s.) nonsignificance in an MWU test. A colored * symbol indicates significance in an MWU test against baseline, that is against the pooled preferences when tested in the presence of the reward (or the absence of the punishment, respectively). Statistical results and source data are in Supplemental Table S1. Other details as in Figure 1.

The opposing effects of PAIRED versus UNPAIRED training conform to the experimental psychology constructs of conditioned excitation versus conditioned inhibition in prediction-error learning rules (e.g., Rescorla and Wagner 1972; Malaka 1999). This view has not remained unchallenged, though. An alternative scenario has been suggested whereby the respective training protocols result in increases versus decreases in how effectively the conditioned stimuli, rather than the reward, are processed (Mackintosh 1975; Pearce and Hall 1980). In the current case, this alternative scenario would suggest that the opposing effects of PAIRED versus UNPAIRED training are due to increased versus decreased attention to the odor. However, our results show that UNPAIRED training not only establishes decreases in odor preference relative to baseline, but also, in particular when three training trials are performed, can establish odor avoidance (Fig. 2C). Such odor avoidance after UNPAIRED training is confirmed in a reanalysis of pertinent previously published data from our laboratory (Fig. 3A–G). These results are incompatible with learned inattention as an explanation for the effects of UNPAIRED training, because a lack of attention may reduce odor preference to zero, but cannot establish avoidance. They thus provide the first demonstration of conditioned inhibition in larval *Drosophila*. We note the variation in baseline odor preferences between the experiments summarized in Figure 3, which arguably reflects variation between, for example, seasons, food, experimental settings, and experimenters even when the same odor and concentration are used (Fig. 1 vs. Fig. 2; Supplemental Fig. S2A–D). This demonstrates the importance of determining the baseline odor preferences separately for each experiment.

**Figure 4.**
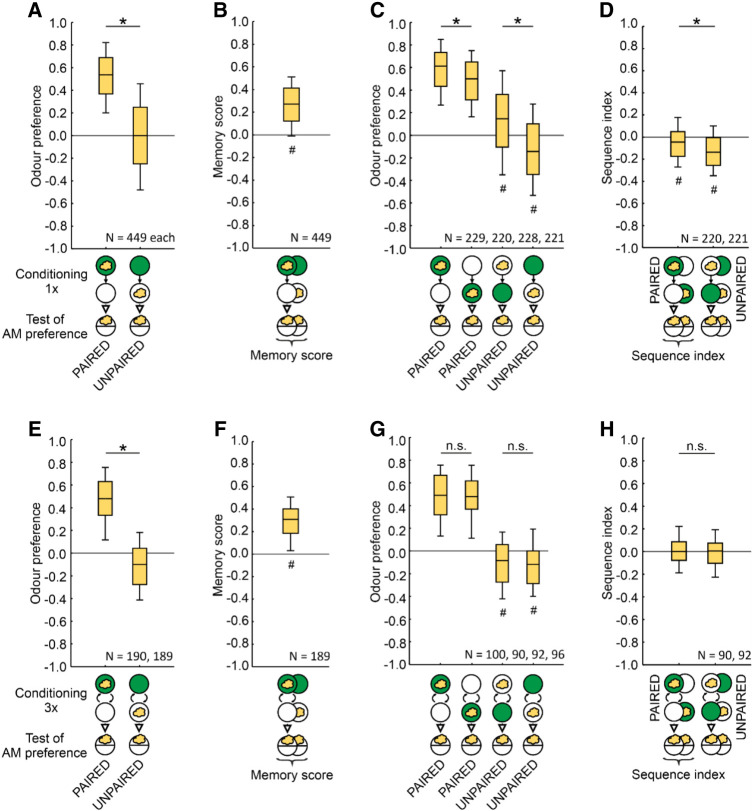
Stronger effect of the sequence of training events for UNPAIRED than for PAIRED training. (*A*,*B*) Larvae received one trial of either PAIRED or UNPAIRED training of odor and the sugar reward. The sequence of events during training was as depicted in half of the cases and the reverse in the other half of the cases. In all cases, training was followed by a test of odor preference, (*A*) which turned out to be higher after PAIRED training than after UNPAIRED training, (*B*) resulting in positive memory scores indicative of appetitive associative memory. (*C*,*D*) Data from *A*, separated by the sequence of events during training. (*C*) Upon PAIRED training, odor preference values are slightly lower when the blank period (open circle) comes first and the odor–reward pairing comes second, as compared to the reverse sequence (two box plots to the *left*). An effect of the sequence of events is also observed upon UNPAIRED training (two box plots to the *right*), with aversion being the result only when the sugar reward is presented first. (*D*) A quantification of the sequence-related differences by the sequence index (Equations 3 and 4) reveals stronger effects of the sequence of training events during UNPAIRED training as compared to PAIRED training. (*E*–*H*) As in *A*–*D*, but for three training trials, providing evidence for associative memory, but no evidence of an effect of the sequence of events during training. (#) Significance to chance level, (*) significance in an MWU test, (n.s.) nonsignificance in an MWU test. Statistical results are given along with source data in the data file Supplemental Table S1. For data separated by individual data set, see Supplemental Figure S2A–D. Other details as in Figure 1.

According to prediction-error learning rules, conditioned excitation ensues when there is a positive prediction error—that is, when, for example, a reward is received although it was not predicted (pleasant surprise). This is straightforward for PAIRED training when the odor is presented together with the reward. Conversely, conditioned inhibition ensues when there is a negative prediction error—that is, when, for example, a predicted reward is not actually received (frustrating surprise). This is not straightforward for UNPAIRED training, however, because one wonders what the source of a reward prediction would be at the moment of odor presentation. Prediction-error learning rules typically make the assumption that presenting the reward alone can establish associative memories for the context (Dweck and Wagner 1970; Rescorla 1972; Bouton and Nelson 1998). When the odor is subsequently presented in that same context, these context–reward associations provide an expectation of reward that, however, is frustratingly not received. The ensuing negative prediction error is then the basis for conditioned inhibition to accrue to the odor. This provides an interesting experimental test because accordingly negative prediction errors would only arise when during UNPAIRED training the reward-only presentation comes first, establishing context–reward associations, and the odor-only presentation comes second. When the odor is presented first, in contrast, there would not yet be any context–reward association that could be frustrated. Such sequence dependence should be particularly prominent when only one training trial is used, because for multiple training trials, the contextual memories established in trial #1 would need to be reckoned with from trial #2 on, effectively “ironing out” sequence effects as UNPAIRED training proceeds. With these considerations in mind, we analyzed two large and mostly unpublished data sets that we have accumulated with course students, interns, scientific guests, and apprentice staff using our standard one-trial training procedure (Fig. 4A–D; total *N* = 898) as well as corresponding experiments with three training trials (Fig. 4E–H; total *N* = 379).

These data sets confirm that odor preference values after one-trial PAIRED training are higher than after UNPAIRED training (Fig. 4A), yielding a positive memory score indicative of appetitive associative memory (Fig. 4B); corresponding results were obtained for the data set using three training trials (Fig. 4E,F). When odor preference values are separated by the sequence of odor and sugar reward presentation during training, it turns out that for the PAIRED case of one-trial training, odor preference values are slightly lower when the blank presentation (EM) comes first and the odor–reward pairing (AM+) comes second as compared to the reverse AM+/EM sequence (Fig. 4C, leftmost two box plots). For the UNPAIRED case, odor preference values are not only lower for the EM+/AM sequence than for the AM/EM+ sequence (Fig. 4C, rightmost two box plots), but—critically important in the argument for conditioned inhibition—show odor avoidance only for the EM+/AM sequence. Compared to the PAIRED case, the sequence of training events has more impact in the UNPAIRED case, as shown by differences in the sequence index (Fig. 4D); this PAIRED–UNPAIRED difference suggests that associative processing plays a role above and beyond the nonassociative processes that impact behavior in either case. No differences in odor preference values between the training sequences were observed upon three-trial training (Fig. 4G,H).

We conclude that the opposing effects of PAIRED versus UNPAIRED training reflect conditioned excitation versus conditioned inhibition, rather than changes in attention.

### No evidence of sensory preconditioning

**Figure 5.**
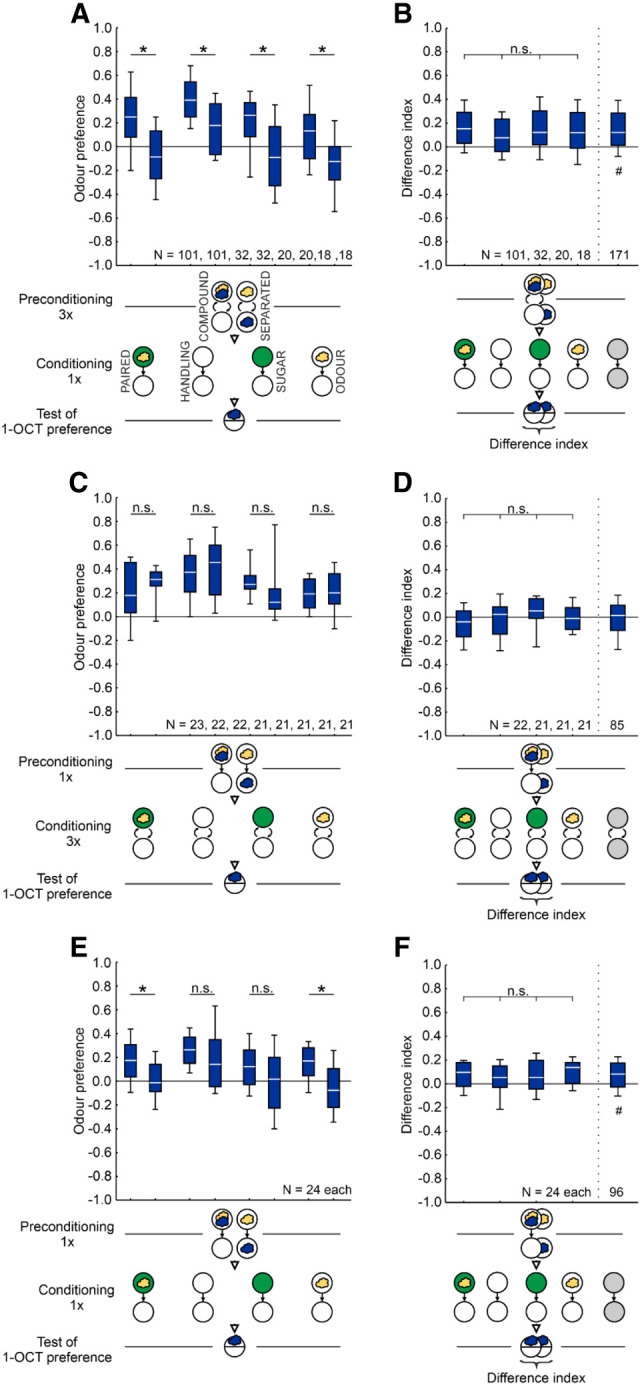
No evidence of sensory preconditioning. (*A*–*F*) During preconditioning the larvae were presented with two odors, AM (yellow cloud) and 1-OCT (blue cloud), either as a COMPOUND on the same Petri dish (white circle) or SEPARATED. During conditioning, the larvae in the experimental groups received PAIRED training of the indicated odor and sugar reward (green circle), whereas larvae in the control groups underwent procedures during which either no odor and no sugar reward (HANDLING), only the SUGAR, or only the ODOR were presented (gray circles represent any of these procedures during conditioning). Subsequently, the larvae were tested for their preference for the odor not used during the conditioning phase (*A*,*C*,*E*), and the difference between the respective COMPOUND and SEPARATED groups was calculated as the difference index (Equation 5) (*B*,*D*,*F*). Sensory preconditioning as an associative phenomenon would be indicated if the difference indices in the experimental, PAIRED group were higher than in the controls. In three variations on the experiment with the number of trials during the preconditioning/conditioning phases set to 3/1 (*A*,*B*), 1/3 (*C*,*D*), or 1/1 (*E*,*F*), no such evidence of sensory preconditioning was observed. Sample sizes for the PAIRED groups in *A*,*B* are high because, unlike all the other experiments in the present study, the control experiments were performed in a staggered, successive manner with the PAIRED groups always run in parallel. (#) Significance from zero in an OSS test, (*) significance in an MWU test or a KW test, (n.s.) nonsignificance in an MWU test or a KW test. Statistical results and source data are in Supplemental Table S1. Other details as in Figure 1.

Larvae first received two odors (AM and 1-OCT) either as a COMPOUND or SEPARATED from each other (preconditioning phase, three trials). For both groups this was followed by PAIRED odor–sugar reward training with one of the odors (AM+) (conditioning phase, one trial) and then by testing with the other odor (1-OCT) (Fig. 5A). We reasoned that if there is sensory preconditioning, then the larvae of the COMPOUND group should show higher odor preference than the larvae of the SEPARATED group, as was indeed the case (Fig. 5A).

However, the same difference in odor preference is observed in control groups for which the treatment in the conditioning phase was varied such that no odor–sugar reward association could be formed (Fig. 5A). Importantly, the difference indices, quantifying the difference in odor preference between the COMPOUND and the SEPARATED group, did not vary across the experimental and control groups (Fig. 5B). Thus, although experiencing the two odors in COMPOUND during preconditioning resulted in consistently higher odor preference during the test than experiencing them SEPARATED from each other, these results do not provide evidence of sensory preconditioning as an associative phenomenon. Varying the number of trials during preconditioning and during conditioning did not provide evidence for sensory preconditioning, either (Fig. 5C–F).

**Figure 6.**
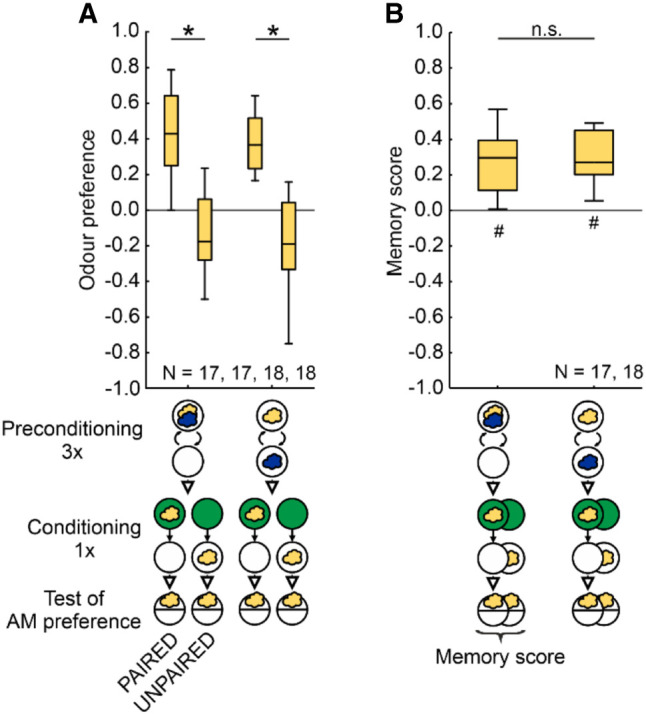
Effective association formation during the conditioning phase. (*A*,*B*) During preconditioning the larvae received the two odors either in compound or separated. This was followed by either PAIRED or UNPAIRED conditioning of the indicated odor and the sugar reward. Subsequently, the larvae were tested for their preference for the odor used during conditioning. (*A*) Regardless of the type of preconditioning, PAIRED training led to higher odor preferences than UNPAIRED training, indicating effective association formation during conditioning. (*B*) Memory scores did not differ between groups, indicating that association formation during conditioning was equally effective regardless of the preconditioning treatment. (#) Significance from zero in an OSS test, (*) significance in an MWU test, (n.s.) nonsignificance in an MWU test. Statistical results and source data are in Supplemental Table S1. All other details as in Figure 5.

Given that the treatment during conditioning did not make any difference in the above experiments, we wondered whether under the current regimen, an association between the odor (AM) and the sugar reward was indeed established during conditioning, and whether it was still behaviorally effective during the test. The larvae first underwent three trials of preconditioning with the two odors presented in COMPOUND or SEPARATED, followed by a single conditioning trial with PAIRED or UNPAIRED training of the odor AM and the sugar reward. We then tested the animals for their AM preferences and found these to be higher after PAIRED than after UNPAIRED training, indicating effective association formation (Fig. 6A) and resulting in positive memory scores (Fig. 6B). Interestingly, the memory scores were not different between the animals that had received the two odors in COMPOUND or SEPARATED during preconditioning, meaning that the type of odor exposure during preconditioning did not affect association formation during the conditioning phase.

Taken together, these results do not offer evidence of sensory preconditioning as an associative phenomenon in larval *Drosophila*.

### No evidence of second-order conditioning

**Figure 7.**
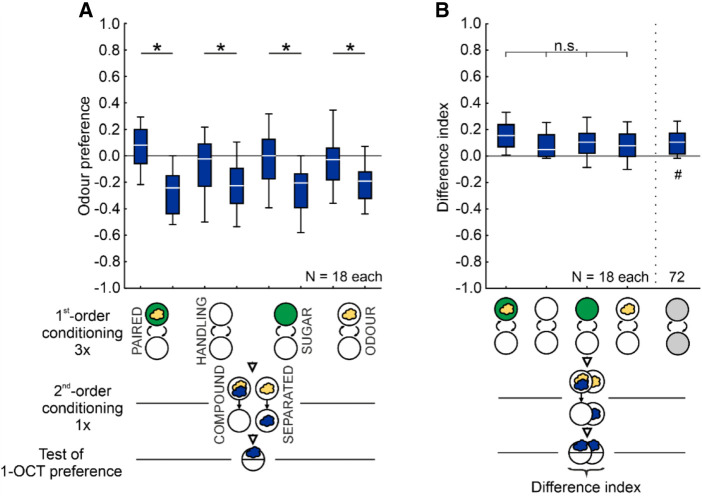
No evidence of second-order conditioning. (*A*,*B*) During first-order conditioning, the larvae in the experimental groups received three trials of PAIRED training of AM (yellow cloud) and a sugar reward, whereas control groups received either no odor and no sugar reward (HANDLING), only the SUGAR, or only the ODOR (gray circles represent any of these procedures during first-order conditioning). In the following, second-order conditioning phase, the larvae were presented with AM either in COMPOUND with or SEPARATED from a novel, “target” odor (1-OCT) (blue cloud). Subsequently, the larvae were tested for their preference for the target odor, and the difference between the respective COMPOUND and SEPARATED groups was calculated as the difference index (Equation 5). Second-order conditioning as an associative phenomenon would be indicated if the difference indices were higher in the PAIRED, experimental groups than in the controls. No evidence for such a result was obtained. (#) Significance to chance level, (*) significance in MWU tests or a KW test, (n.s.) nonsignificance in MWU tests or a KW test. Statistical results and source data are in Supplemental Table S1. All other details as in Figure 5.

Larvae in the experimental groups first underwent conditioning to establish an odor–reward association (first-order conditioning, three trials). This was followed by a second-order conditioning phase in which the previously rewarded odor was presented either in COMPOUND with or SEPARATED from a novel, target odor (one trial). Then the larvae were tested for their preference for the target odor (Fig. 7). We reasoned that if there is second-order conditioning, then the larvae of the COMPOUND group should show a higher odor preference than the larvae of the SEPARATED group, as was indeed the case (Fig. 7A). However, the same difference in odor preference is observed in control groups for which the treatment during first-order conditioning was varied such that no odor–sugar reward association could be formed (Fig. 7A). Importantly, the difference indices did not vary across the experimental and control groups (Fig. 7B). These results do not provide evidence of second-order conditioning as an associative phenomenon (preliminary experiments in the aversive domain, using a modified protocol and associations between odor and electric shock during first-order conditioning, likewise yielded no such evidence: Supplemental Fig. S3A–D; Saumweber et al. 2011; Tomasiunaite et al. 2018).

**Figure 8.**
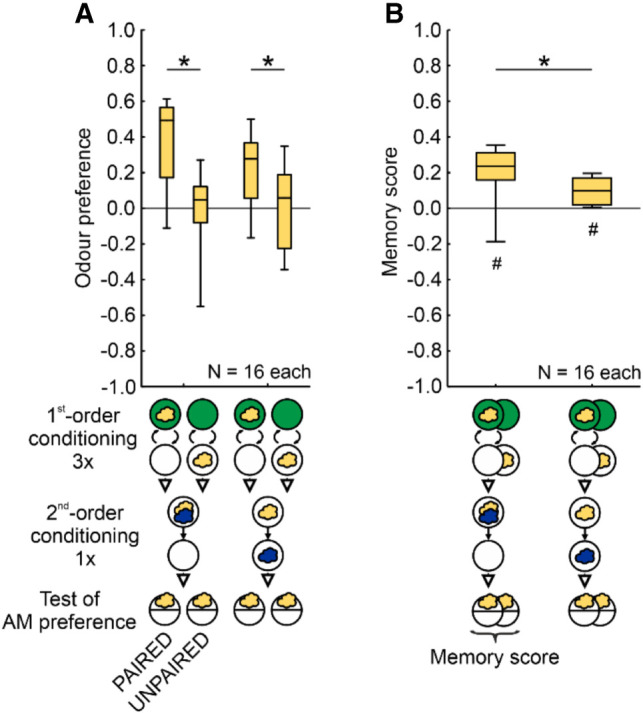
Effective first-order associations. (*A*,*B*) Larvae received three trials of first-order conditioning with either PAIRED or UNPAIRED training of AM (yellow cloud) and a sugar reward. This was followed by presenting AM either in a compound with or separated from 1-OCT (blue cloud) as the target odor, followed by testing for the preference for AM as the first-order conditioning odor. Regardless of the type of odor presentation in the second experimental phase, PAIRED training led to higher odor preferences than UNPAIRED training, resulting in positive memory scores in both cases and indicating effective first-order conditioning associations. In comparison to the compound case, the memory scores were less when the two odors were presented separately, though, arguing for more effective extinction learning when the first-order conditioning odor is experienced alone. (#) Significance to chance level, (*) significance in an MWU test. Statistical results and source data are in Supplemental Table S1. All other details as in Figure 5.

To test for the possibility that the treatments in the second phase of the experiment rendered the first-order association ineffective, we conducted a control experiment in which the larvae were trained either PAIRED or UNPAIRED with the first-order conditioning odor (AM) and a sugar reward, followed by the presentation of the first-order conditioning odor either in COMPOUND with or SEPARATED from the target odor (1-OCT). This was followed by testing for the preference for the first-order conditioning odor (Fig. 8A). PAIRED training with the first-order conditioning odor yielded a higher preference for it than UNPAIRED training with it, both when it was subsequently presented in COMPOUND with and SEPARATED from the target odor (Fig. 8A). Accordingly, appetitive associative memory was observed in both cases (Fig. 8B), showing that the effects of first-order conditioning were not rendered ineffective by the treatments in the second experimental phase. We note that, when compared to the COMPOUND case, the memory scores were less when the two odors were presented SEPARATED in the second-order conditioning phase; it must remain unresolved for now whether this reflects more effective extinction learning when the first-order conditioning odor is experienced alone. In any event, the conclusion remains that from the present results, there is no evidence of second-order conditioning as an associative phenomenon in larval *Drosophila*.

## Discussion

We provide evidence for conditioned inhibition in larval *Drosophila*, corresponding to what has been reported in adult flies (Barth et al. 2014; Jacob and Waddell 2020) and honeybees (Takeda 1961; Bitterman et al. 1983; Hellstern et al. 1998; Chandra et al. 2010; Matsumoto et al. 2012; Mahoney et al. 2024). However, our data do not provide evidence for sensory preconditioning or second-order conditioning in larval *Drosophila*. This does not amount to evidence of their absence, though. Changes to the experimental protocol that could uncover these forms of learning in larval *Drosophila* include changes to the number or temporal spacing of trials, the use of different odor concentrations or of serial rather than simultaneous odor compounds, different odors or combinations of odors with visual stimuli, or other kinds of the reinforcer. Also, more fine-grained analyses of behavior through video tracking might reveal the effects of training that the current study has overlooked. It is expressly only with these caveats in mind that the following discussion supposes that larval *Drosophila* are not capable of sensory preconditioning and second-order conditioning. Why not?

### Ways of life

Sensory preconditioning and second-order conditioning have been demonstrated in adult flies (Brembs and Heisenberg 2001; Guo and Guo 2005; Tabone and De Belle 2011; Martinez-Cervantes et al. 2022; Yamada et al. 2023) and honeybees (Takeda 1961; Bitterman et al. 1983; Müller et al. 2000; Hussaini et al. 2007), including with protocols that use binary odor compounds. In contrast, we report an absence of evidence for these forms of learning in larval *Drosophila*. Which of the differences in the biology of larval *Drosophila* versus adult flies and honeybees could account for this discrepancy?

Insect larvae are sexually undifferentiated and dedicated to feeding and growth. Indeed, the salivary glands of larval *Drosophila* are larger than their brain. The caste of honeybee used in the above-cited experiments is likewise asexual in motivation and dedicated to foraging. In motivational terms, therefore, larval *Drosophila* seem closer to honeybees than to adult flies.

What differentiates larval *Drosophila* from adult flies and honeybees is rather that the larvae live on or in decaying fruit as their “food-home.” They thus do not need to bother much with finding as-yet absent resources such as food, a home (as is the case for honeybees), or mating partners (as adult flies need to do). It would seem that larvae can, therefore, organize their behavior in a more “online” manner, that is in relation to physically present resources. For adult flies and honeybees, in contrast, the behavior needs to be organized in a more “offline” manner, toward desired but as-yet physically absent goals. As argued in the introductory section, sensory preconditioning and second-order conditioning both require chained processing in relation to physically absent cues or reinforcement and thus chained “offline” processing. These tasks might, therefore, tap into cognitive abilities evolved for a life as lived by adult flies and honeybees.

### Brain organization

**Figure 9.**
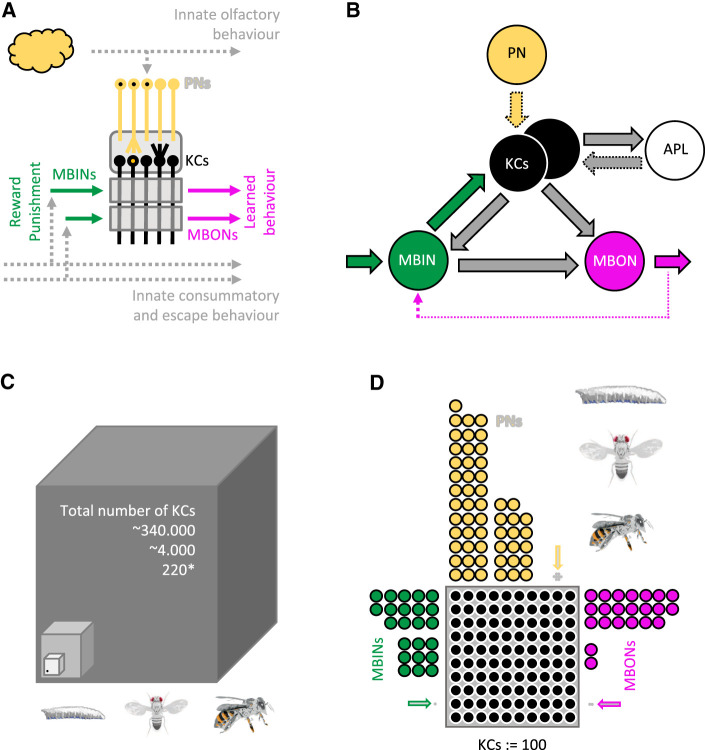
Simplified organization of the mushroom body. (*A*) The mushroom body intrinsic neurons (Kenyon cells [KCs]; black) form a sparse, combinatorial representation of odors, established through a divergence/convergence connectivity with sensory projection neurons (PNs; yellow) in the calyx (rounded gray rectangle). Axonal projections of the KCs pass through compartments (gray rectangles) established by axonal branches of modulatory mushroom body input neurons (MBINs; green) (most of these are dopaminergic) and dendritic branches of mushroom body output neurons (MBONs; pink). Of a total of 10 compartments, one with a rewarding and one with a punishing MBIN is shown. Associative memories are established through compartmentally specific plasticity at the synapses between odor-activated KCs and MBONs. This changes the balance of activity in avoidance- versus approach-promoting MBONs underlying learned behavior. Pathways for innate behavior are shown in gray. (*B*) PN → KC synapses are found only in the input region of the mushroom body (the calyx), but not in the lobe compartments (stippled yellow arrow). In larval *Drosophila*, APL → KC synapses are found only in the calyx (stippled gray arrow), rather than throughout the mushroom body as in adult flies; also, only six out of 10 compartments, rather than all compartments as in adult flies, feature KC → APL synapses. The stippled pink arrow indicates mostly indirect feedback from MBONs to MBINs. Innervations by more than one MBIN or MBON, multiple-compartment MBINs and MBONs, and lateral connections between MBONs as well as between KCs are not shown. (*C*,*D*) Cell numbers in the mushroom bodies of larval *Drosophila*, adult flies, and honeybees summed for both hemispheres. Shown in *C* are the numbers of KCs (the dot indicates a single KC within a cube of 6 × 6 × 6 KCs), and in *D* the number of the indicated cell types normalized to the number of KCs. Numbers can be found in Supplemental Table S1 and are based on Eichler et al. (2017) and Saumweber et al. (2018) for a first-instar *Drosophila* larva, and on Bates et al. (2020), Li et al. (2020), and Schlegel et al. (2021) for adult flies. For third-instar larvae as used in the present behavioral experiments, the number of KCs is ∼600–800 (*). Regarding honeybees, our estimations are based on Witthöft (1967), Schäfer and Rehder (1989), Maronde (1991), Rybak and Menzel (1993), Kreissl et al. (1994), Rybak (1994), Stevenson and Spörhase-Eichmann (1995), Grünewald (1999), Gronenberg (2001), Schröter and Menzel (2003), Schröter et al. (2007), and Rybak (2011), as well as Zwaka et al. (2018).

The brains of larval *Drosophila*, adult flies, honeybees, and indeed insects more generally are organized in largely the same way, including the mushroom body, a central-brain structure serving olfactory associative memory (for a simplified overview, see Fig. 9A,B). It, therefore, seems that the relevant differences in brain organization are not to be found in the principles but in the details (Menzel 2013). Which ones?

*Central complex*. Although the brain of larval *Drosophila* is organized according to the same principles as in adult flies and honeybees, the numbers of neurons are reduced. Indeed, the central complex comprises so few neurons in the case of larval *Drosophila* that it cannot be recognized as a histological structure but only from the circuit motifs that these neurons establish. Central complex function underlies course control relative to local sensory cues during the pursuit of as-yet absent goals (Pfeiffer 2023)—for example, when honeybees navigate between their hive and flower patches way beyond the catchment area of their sensory systems. To the extent that a more sophisticated organization of behavior in relation to absent goals is required for sensory preconditioning and second-order conditioning, the performance of these tasks might, therefore, come more easily to adult flies and honeybees with their more elaborate central complex.

*Ascending olfactory pathways*. In comparison to adult flies and honeybees, the ascending olfactory pathways in larval *Drosophila* are characterized by lower numbers of expressed olfactory receptor genes, sensory neurons, and PNs and a lack of cellular redundancy at these layers of processing, as well as by less interhemispheric communication, by a lower number of mushroom body intrinsic neurons (called KCs), and by the fact that about one-fifth of these KCs receive input from only a single PN in what appears to be a labeled-line sensory-motor pathway. This suggests that their ascending olfactory system has a substantially poorer signal-to-noise ratio and provides a much less nuanced representation of odors than in adult flies and honeybees. The present protocols for sensory preconditioning and second-order conditioning might thus be too demanding, as they require an ability to process binary odor compounds by their elements (something of which larvae are in principle capable, though; Chen and Gerber 2014; Chen et al. 2017) and/or a process of pattern completion when only one of these elements is physically present.

*Mushroom body*. The number of KCs is relatively similar in larval *Drosophila* and adult flies, at least in comparison to the much higher number in honeybees (Fig. 9C). Consideration of the number of PNs and the number of modulatory MBINs *relative to the number of KCs* paints a similar picture (Fig. 9D). The relative number of MBONs does not set larval *Drosophila* against adult flies and honeybees, either.

*APL and DPM neurons*. The mushroom body of *Drosophila* features a giant intrinsic neuron called APL (Mancini et al. 2023, and references therein). This GABAergic neuron is embryonically born, persists through metamorphosis, reciprocally connects to KCs, and functionally corresponds to the GABAergic A3 neurons in honeybees (Rybak and Menzel 1993). In adult flies such reciprocal connectivity is observed throughout the mushroom body (as is the case for the KCs and GABAergic neurons in honeybees; Ganeshina and Menzel 2001; Zwaka et al. 2018). In larval *Drosophila*, however, reciprocal APL ←→ KC connectivity is only found in the calyx region of the mushroom body, where the signals from the PNs are received. In the lobe regions of the larval mushroom body, where all but two of the MBONs are located and where behavioral output is instructed, APL → KC connections are conspicuously absent. It would thus seem that in adult flies APL and, in the case of honeybees, the A3_LC_ neurons (Zwaka et al. 2018) can prevent KC → MBON signaling and thus “take KCs offline” from behavioral control, whereas this would not be possible in larval *Drosophila* (for the significance of such offline processing for action planning and cognition, see Menzel 2013). Interestingly, lobe-to-calyx feedback, possible through APL in larval *Drosophila* and adult flies and through the A3_FB_ neurons in honeybees (Zwaka et al. 2018), was suggested to underlie reversal learning (Boitard et al. 2015)—a faculty indeed observed in all three kinds of animal (Tully and Quinn 1985; Boitard et al. 2015; Mancini et al. 2019). In addition, in adult flies APL is electrically coupled to the DPM neuron, which is absent in larval *Drosophila*, and concerted APL–DPM action has been reported to moderate KC–KC communication (Okray et al. 2023) in a process that should support pattern completion (Cayco-Gajic and Silver 2019). To the extent that mushroom body function “offline” from behavioral control and/or pattern completion is involved in sensory preconditioning and second-order conditioning, the absence of these forms of learning in larval *Drosophila* could thus be related to differences in APL organization or the absence of DPM.

There thus seem to be four aspects of brain organization in larval *Drosophila* versus adult flies and honeybees that have the potential to explain the absence of sensory preconditioning and second-order conditioning in larvae: (i) an insufficiently differentiated central complex; (ii) an insufficient signal-to-noise ratio in the ascending olfactory pathways and mushroom bodies, and thus an insufficiently nuanced representation of odor mixtures; (iii) the absence of APL → KC connections in the mushroom body output region; and (iv) the absence of the DPM neuron. We note the mnemonic faculties that have nevertheless been observed in larval *Drosophila*, including discrimination, generalization, reversal learning, memory consolidation, an adaptive dominance of consummatory over learned behavior (Gerber and Hendel 2006; Mishra et al. 2010; Schleyer et al. 2011, 2015a,b; Widmann et al. 2016; Mancini et al. 2019), and conditioned inhibition (this study).

### Models

Conditioned inhibition through unpaired training of a cue with a reward can be accommodated by prediction-error learning rules (Malaka 1999 and references therein) (for a model inspired by the functional anatomy of the mushroom body not involving prediction errors, see Gkanias et al. 2022). These models require that at the moment of presenting the cue, there is a negative prediction error, as is the case when a predicted reward, frustratingly, is not received. This raises the question of the source of the reward prediction. Two nonexclusive scenarios may be invoked. A reward prediction may arise from previously established context–reward associations (Rescorla and Wagner 1972) or from the generalization of previously established cue–reward associations (Jürgensen et al. 2024). Our observation that unpaired training does not lead to odor avoidance when there is no possibility for context–reward associations to have been established (Fig. 4C, second box plot from the right) supports the contextual scenario.

Sensory preconditioning, however, is beyond the scope of the aforementioned models because it takes place between two cues rather than between a cue and reinforcement. What is required for sensory preconditioning is a process that endows two cues, based on their past co-occurrence, with the capacity to call up each other's representation when either is presented alone. In analogy to pattern completion in the cerebellum and the hippocampus (Rudy and Sutherland 1995; Rolls 2013; Cayco-Gajic and Silver 2019), this could be achieved by modulations of KC–KC signaling (Müller et al. 2000; Okray et al. 2023). It would be interesting to see whether models of the mushroom body that either do or do not feature the possibility of modulations in KC–KC signaling yield sensory preconditioning.

To account for second-order conditioning was one of the main goals in the development of real-time models of reinforcement learning through prediction errors (Malaka 1999 and references therein) (for an alternative scenario not involving prediction errors, see Gkanias et al. 2022). What most prediction-error models have in common is that by virtue of the A+ association established in phase (i) cue A can call up a representation of +. During AB presentations in phase (ii) this associatively activated representation of + can be associated with B. The resulting B+ association is thus the immediate cause of the response to B during the test. Given that a subset of dopaminergic mushroom body input neurons (MBINs of the DAN type) is thought to mediate internal reward signals, this raises the question of how a learned cue A can activate DANs. As associative learning entails plasticity at the KC → MBON synapses, this could happen through feedback from the MBONs to the DANs. Such feedback was recently shown by Yamada et al. (2023) to underlie second-order conditioning in adult flies (see also König et al. 2019) and is compatible with the functional anatomy of the mushroom body in larval *Drosophila* as well (Eschbach et al. 2020). But why, then, is second-order conditioning not experimentally observed in the case of the larva?

Modeling studies typically restrict themselves to the task-relevant pathways. For example, they consider the ascending olfactory and gustatory pathways, the mushroom body, and the first steps of the mushroom body efferent circuits. However, what goes on in the “rest” of the animal is typically ignored, for example, in relation to the visual, thermo-, mechano-, or hygrosensory pathways, in relation to processing in the central complex or the ventral nerve cord, or in relation to signaling between the brain, gut, and glands (for an exception, see Sakagiannis et al. 2021). The low number of neurons and the absence of cellular redundancy in larval *Drosophila* possibly makes them more susceptible to such influences than adult flies or honeybees. It would, therefore, be interesting to challenge models of the mushroom body that use numbers of model neurons corresponding to larval *Drosophila* versus adult flies and honeybees with various levels of noise to see how this affects first- and second-order conditioning.

We note that there is an alternative explanation of second-order conditioning—namely, that it is a phenomenon of memory retrieval (Savastano and Miller 1998; Molet et al. 2012). It is suggested that during phase (ii) an AB association is formed and that through this AB association, *at the moment of the test*, B calls up A, which then calls up +. In other words, the immediate cause of the response is the A+ association established in phase (i), not a B+ association established in phase (ii). Such a retrieval account of second-order conditioning has not so far been considered in models of the insect brain.

In closing, we would like to argue that when it comes to identifying cognitive limits, demonstrations of the absence of a cognitive faculty are a success, because limits can only be determined through a failure to transgress them.

### Competing interest statement

The authors declare no competing interests.

## Supplementary Material

Supplement 1

Supplement 2

## References

[LM053726SENC1] Aceves-Piña EO, Quinn WG. 1979. Learning in normal and mutant *Drosophila* larvae. Science 206: 93–96. 10.1126/science.206.4414.93 17812455

[LM053726SENC2] Barth J, Dipt S, Pech U, Hermann M, Riemensperger T, Fiala A. 2014. Differential associative training enhances olfactory acuity in *Drosophila melanogaster*. J Neurosci 34: 1819–1837. 10.1523/JNEUROSCI.2598-13.2014 24478363 PMC6827587

[LM053726SENC3] Bates AS, Schlegel P, Roberts RJV, Drummond N, Tamimi IFM, Turnbull R, Zhao X, Marin EC, Popovici PD, Dhawan S, 2020. Complete connectomic reconstruction of olfactory projection neurons in the fly brain. Curr Biol 30: 3183–3199. 10.1016/j.cub.2020.06.042 32619485 PMC7443706

[LM053726SENC4] Bitterman ME, Menzel R, Fietz A, Schäfer S. 1983. Classical conditioning of proboscis extension in honeybees (*Apis mellifera*). J Comp Psychol 97: 107–119. 10.1037/0735-7036.97.2.107 6872507

[LM053726SENC5] Boitard C, Devaud J-M, Isabel G, Giurfa M. 2015. GABAergic feedback signaling into the calyces of the mushroom bodies enables olfactory reversal learning in honey bees. Front Behav Neurosci 9: 198. 10.3389/fnbeh.2015.00198 26283938 PMC4518197

[LM053726SENC6] Boto T, Stahl A, Tomchik SM. 2020. Cellular and circuit mechanisms of olfactory associative learning in *Drosophila*. J Neurogenet 34: 36–46. 10.1080/01677063.2020.1715971 32043414 PMC7147969

[LM053726SENC7] Bouton ME, Nelson JB. 1998. The role of context in classical conditioning: some implications for cognitive behavior therapy. In Learning theory and behavior therapy (ed. O'Donohue WT), pp. 59–84. Allyn & Bacon, Boston.

[LM053726SENC8] Brand AH, Perrimon N. 1993. Targeted gene expression as a means of altering cell fates and generating dominant phenotypes. Development 118: 401–415. 10.1242/dev.118.2.401 8223268

[LM053726SENC9] Brembs B, Heisenberg M. 2001. Conditioning with compound stimuli in *Drosophila melanogaster* in the flight simulator. J Exp Biol 204: 2849–2859. 10.1242/jeb.204.16.2849 11683440

[LM053726SENC10] Brogden WJ. 1939. Sensory pre-conditioning. J Exp Psychol 25: 323–332. 10.1037/h0058944 18920626

[LM053726SENC11] Cayco-Gajic NA, Silver RA. 2019. Re-evaluating circuit mechanisms underlying pattern separation. Neuron 101: 584–602. 10.1016/j.neuron.2019.01.044 30790539 PMC7028396

[LM053726SENC12] Chandra SBC, Wright GA, Smith BH. 2010. Latent inhibition in the honey bee, *Apis mellifera*: is it a unitary phenomenon? Anim Cogn 13: 805–815. 10.1007/s10071-010-0329-6 20521073

[LM053726SENC13] Chen YC, Gerber B. 2014. Generalization and discrimination tasks yield concordant measures of perceived distance between odours and their binary mixtures in larval *Drosophila*. J Exp Biol 217: 2071–2077. 10.1242/jeb.100966 24920835 PMC4191342

[LM053726SENC14] Chen Y, Mishra D, Gläß S, Gerber B. 2017. Behavioral evidence for enhanced processing of the minor component of binary odor mixtures in larval *Drosophila*. Front Psychol 8: 1923. 10.3389/fpsyg.2017.01923 29163299 PMC5672140

[LM053726SENC15] Cognigni P, Felsenberg J, Waddell S. 2018. Do the right thing: neural network mechanisms of memory formation, expression and update in *Drosophila*. Curr Opin Neurobiol 49: 51–58. 10.1016/j.conb.2017.12.002 29258011 PMC5981003

[LM053726SENC16] Diegelmann S, Klagges B, Michels B, Schleyer M, Gerber B. 2013. Maggot learning and synapsin function. J Exp Biol 216: 939–951. 10.1242/jeb.076208 23447663

[LM053726SENC17] Dudai Y, Jan YN, Byers D, Quinn WG, Benzer S. 1976. dunce, a mutant of *Drosophila* deficient in learning. Proc Natl Acad Sci 73: 1684–1688. 10.1073/pnas.73.5.1684 818641 PMC430364

[LM053726SENC18] Dweck CS, Wagner AR. 1970. Situational cues and correlation between CS and US as determinants of the conditioned emotional response. Psychon Sci 18: 145–147. 10.3758/BF03332345

[LM053726SENC19] Eichler K, Li F, Litwin-Kumar A, Park Y, Andrade I, Schneider-Mizell CM, Saumweber T, Huser A, Eschbach C, Gerber B, 2017. The complete connectome of a learning and memory centre in an insect brain. Nature 548: 175–182. 10.1038/nature23455 28796202 PMC5806122

[LM053726SENC20] El-Keredy A, Schleyer M, König C, Ekim A, Gerber B. 2012. Behavioural analyses of quinine processing in choice, feeding and learning of larval *Drosophila*. PLoS ONE 7: e40525. 10.1371/journal.pone.0040525 22802964 PMC3393658

[LM053726SENC21] Eschbach C, Zlatic M. 2020. Useful road maps: studying *Drosophila* larva's central nervous system with the help of connectomics. Curr Opin Neurobiol 65: 129–137. 10.1016/j.conb.2020.09.008 33242722 PMC7773133

[LM053726SENC22] Eschbach C, Fushiki A, Winding M, Schneider-Mizell CM, Shao M, Arruda R, Eichler K, Valdes-Aleman J, Ohyama T, Thum AS, 2020. Recurrent architecture for adaptive regulation of learning in the insect brain. Nat Neurosci 23: 544–555. 10.1038/s41593-020-0607-9 32203499 PMC7145459

[LM053726SENC23] Ganeshina O, Menzel R. 2001. GABA-immunoreactive neurons in the mushroom bodies of the honeybee: an electron microscopic study. J Comp Neurol 437: 335–349. 10.1002/cne.1287 11494260

[LM053726SENC24] Gerber B, Aso Y. 2017. Localization, diversity, and behavioral expression of associative engrams in *Drosophila*. In Learning and memory. A comprehensive reference (ed. Byrne JH), pp. 463–473. Elsevier, Oxford.

[LM053726SENC25] Gerber B, Hendel T. 2006. Outcome expectations drive learned behaviour in larval *Drosophila*. Proc R Soc B Biol Sci 273: 2965–2968. 10.1098/rspb.2006.3673 PMC1639518 17015355

[LM053726SENC26] Gerber B, Stocker RF. 2007. The *Drosophila* larva as a model for studying chemosensation and chemosensory learning: a review. Chem Senses 32: 65–89. 10.1093/chemse/bjl030 17071942

[LM053726SENC27] Gkanias E, McCurdy LY, Nitabach MN, Webb B. 2022. An incentive circuit for memory dynamics in the mushroom body of *Drosophila melanogaster*. eLife 11: e75611. 10.7554/eLife.75611 35363138 PMC8975552

[LM053726SENC28] Gronenberg W. 2001. Subdivisions of hymenopteran mushroom body calyces by their afferent supply. J Comp Neurol 435: 474–489. 10.1002/cne.1045 11406827

[LM053726SENC29] Grünewald B. 1999. Morphology of feedback neurons in the mushroom body of the honeybee, *Apis mellifera*. J Comp Neurol 404: 114–126. 10.1002/(SICI)1096-9861(19990201)404:1<114::AID-CNE9>3.0.CO;2-# 9886029

[LM053726SENC30] Guo J, Guo A. 2005. Crossmodal interactions between olfactory and visual learning in *Drosophila*. Science 309: 307–310. 10.1126/science.1111280 16002621

[LM053726SENC31] Guven-Ozkan T, Davis RL. 2014. Functional neuroanatomy of *Drosophila* olfactory memory formation. Learn Mem 21: 519–526. 10.1101/lm.034363.114 25225297 PMC4175493

[LM053726SENC32] Heisenberg M, Borst A, Wagner S, Byers D. 1985. *Drosophila* mushroom body mutants are deficient in olfactory learning. J Neurogenet 2: 1–30. 10.3109/01677068509100140 4020527

[LM053726SENC33] Hellstern F, Malaka R, Hammer M. 1998. Backward inhibitory learning in honeybees: a behavioral analysis of reinforcement processing. Learn Mem 4: 429–444. 10.1101/lm.4.5.429 10701882

[LM053726SENC34] Holm S. 1979. A simple sequentially rejective multiple test procedure. Scand J Stat 6: 65–70.

[LM053726SENC35] Hussaini SA, Komischke B, Menzel R, Lachnit H. 2007. Forward and backward second-order Pavlovian conditioning in honeybees. Learn Mem 14: 678–683. 10.1101/lm.471307 17911371 PMC2044558

[LM053726SENC36] Jacob PF, Waddell S. 2020. Spaced training forms complementary long-term memories of opposite valence in *Drosophila*. Neuron 106: 977–991. 10.1016/j.neuron.2020.03.013 32289250 PMC7302427

[LM053726SENC37] Jürgensen A-M, Sakagiannis P, Schleyer M, Gerber B, Nawrot MP. 2024. Prediction error drives associative learning and conditioned behavior in a spiking model of *Drosophila* larva. *iScience* **27:** 108640. 10.1016/j.isci.2023.108640 PMC10824792 38292165

[LM053726SENC38] König C, Khalili A, Niewalda T, Gao S, Gerber B. 2019. An optogenetic analogue of second-order reinforcement in *Drosophila*. Biol Lett 15: 20190084. 10.1098/rsbl.2019.0084 31266421 PMC6684970

[LM053726SENC39] Kreissl S, Eichmüller S, Bicker G, Rapus J, Eckert M. 1994. Octopamine-like immunoreactivity in the brain and subesophageal ganglion of the honeybee. J Comp Neurol 348: 583–595. 10.1002/cne.903480408 7530730

[LM053726SENC40] Li HH, Kroll JR, Lennox SM, Ogundeyi O, Jeter J, Depasquale G, Truman JW. 2014. A GAL4 driver resource for developmental and behavioral studies on the larval CNS of *Drosophila*. Cell Rep 8: 897–908. 10.1016/j.celrep.2014.06.065 25088417

[LM053726SENC41] Li F, Lindsey J, Marin EC, Otto N, Dreher M, Dempsey G, Stark I, Bates AS, Pleijzier MW, Schlegel P, 2020. The connectome of the adult *Drosophila* mushroom body provides insights into function. eLife 9: e62576. 10.7554/eLife.62576 33315010 PMC7909955

[LM053726SENC42] Mackintosh NJ. 1975. A theory of attention: variations in the associability of stimuli with reinforcement. Psychol Rev 82: 276–298. 10.1037/h0076778

[LM053726SENC43] Mahoney S, Hosler J, Smith BH. 2024. Reinforcement expectation in the honey bee (*Apis mellifera*): Can downshifts in reinforcement show conditioned inhibition? bioRxiv 10.1101/2024.01.03.574098 PMC11199939 38862176

[LM053726SENC44] Malaka R. 1999. Models of classical conditioning. Bull Math Biol 61: 33–83. 10.1006/bulm.1998.9998

[LM053726SENC45] Mancini N, Hranova S, Weber J, Weiglein A, Schleyer M, Weber D, Thum AS, Gerber B. 2019. Reversal learning in *Drosophila* larvae. Learn Mem 26: 424–435. 10.1101/lm.049510.119 31615854 PMC6796787

[LM053726SENC46] Mancini N, Thoener J, Tafani E, Pauls D, Mayseless O, Strauch M, Eichler K, Champion A, Kobler O, Weber D, 2023. Rewarding capacity of optogenetically activating a giant GABAergic central-brain interneuron in larval *Drosophila*. J Neurosci 43: 7393–7428. 10.1523/JNEUROSCI.2310-22.2023 37734947 PMC10621887

[LM053726SENC47] Maronde U. 1991. Common projection areas of antennal and visual pathways in the honeybee brain, *Apis mellifera*. J Comp Neurol 309: 328–340. 10.1002/cne.903090304 1918441

[LM053726SENC48] Martinez-Cervantes J, Shah P, Phan A, Cervantes-Sandoval I. 2022. Higher-order unimodal olfactory sensory preconditioning in *Drosophila*. eLife 11: e79107. 10.7554/eLife.79107 36129180 PMC9566850

[LM053726SENC49] Matsumoto Y, Menzel R, Sandoz J-C, Giurfa M. 2012. Revisiting olfactory classical conditioning of the proboscis extension response in honey bees: a step toward standardized procedures. J Neurosci Methods 211: 159–167. 10.1016/j.jneumeth.2012.08.018 22960052

[LM053726SENC50] Menzel R. 2013. Learning, memory, and cognition: animal perspectives. In Neurosciences—from molecule to behavior: a university textbook (ed. Galizia CG, Lledo PM), pp. 629–653. Springer, Berlin.

[LM053726SENC51] Menzel R, Benjamin P. (eds.) 2013. Invertebrate learning and memory. Elsevier Academic, Amsterdam.

[LM053726SENC52] Michels B, Saumweber T, Biernacki R, Thum J, Glasgow RDV, Schleyer M, Chen YC, Eschbach C, Stocker RF, Toshima N, 2017. Pavlovian conditioning of larval *Drosophila*: an illustrated, multilingual, hands-on manual for odor–taste associative learning in maggots. Front Behav Neurosci 11: 45. 10.3389/fnbeh.2017.00045 PMC5395560 28469564

[LM053726SENC53] Mishra D, Louis M, Gerber B. 2010. Adaptive adjustment of the generalization-discrimination balance in larval *Drosophila*. J Neurogenet 24: 168–175. 10.3109/01677063.2010.498066 20807100

[LM053726SENC54] Molet M, Miguez G, Cham HX, Miller RR. 2012. When does integration of independently acquired temporal relationships take place? J Exp Psychol Anim Behav Process 38: 369–380. 10.1037/a0029379 22905828

[LM053726SENC55] Müller D, Gerber B, Hellstern F, Hammer M, Menzel R. 2000. Sensory preconditioning in honeybees. J Exp Biol 203: 1351–1364. 10.1242/jeb.203.8.1351 10729283

[LM053726SENC56] Neuser K, Husse J, Stock P, Gerber B. 2005. Appetitive olfactory learning in *Drosophila* larvae: effects of repetition, reward strength, age, gender, assay type and memory span. Anim Behav 69: 891–898. 10.1016/j.anbehav.2004.06.013

[LM053726SENC057] Niewalda T, Singhal N, Fiala A, Saumweber T, Wegener S, Gerber B. 2008. Salt processing in larval *Drosophila*: Choice, feeding, and learning shift from appetitive to aversive in a concentration-dependent way. Chem Sens 33: 685–692. 10.1093/chemse/bjn037 PMC2565773 18640967

[LM053726SENC57] Okray Z, Jacob PF, Stern C, Desmond K, Otto N, Talbot CB, Vargas-Gutierrez P, Waddell S. 2023. Multisensory learning binds neurons into a cross-modal memory engram. Nature 617: 777–784. 10.1038/s41586-023-06013-8 37100911 PMC10208976

[LM053726SENC58] Paisios E, Rjosk A, Pamir E, Schleyer M. 2017. Common microbehavioral “footprint” of two distinct classes of conditioned aversion. Learn Mem 24: 191–198. 10.1101/lm.045062.117 28416630 PMC5397685

[LM053726SENC59] Pavlov IP. 1927. Conditioned reflexes: an investigation of the physiological activity of the cerebral cortex. Oxford University Press, Oxford. 10.5214/ans.0972-7531.1017309 PMC4116985 25205891

[LM053726SENC60] Pearce JM, Hall G. 1980. A model for Pavlovian learning: variations in the effectiveness of conditioned but not of unconditioned stimuli. Psychol Rev 87: 532–552. 10.1037/0033-295X.87.6.532 7443916

[LM053726SENC61] Pfeiffer K. 2023. The neuronal building blocks of the navigational toolkit in the central complex of insects. Curr Opin Insect Sci 55: 100972. 10.1016/j.cois.2022.100972 36126877

[LM053726SENC62] Pfeiffer BD, Ngo T-TB, Hibbard KL, Murphy C, Jenett A, Truman JW, Rubin GM. 2010. Refinement of tools for targeted gene expression in *Drosophila*. Genetics 186: 735–755. 10.1534/genetics.110.119917 20697123 PMC2942869

[LM053726SENC63] Rescorla RA. 1969. Pavlovian conditioned inhibition. Psychol Bull 72: 77–94. 10.1037/h0027760

[LM053726SENC64] Rescorla RA. 1972. Informational variables in Pavlovian conditioning. In Psychology of learning and motivation (ed. Bower GH), Vol. 6, pp. 1–46. Academic, New York.

[LM053726SENC65] Rescorla RA. 1980. Pavlovian second-order conditioning: studies in associative learning. Lawrence Erlbaum Associates, Hillsdale, NJ.

[LM053726SENC66] Rescorla RA. 1988a. Behavioral studies of Pavlovian conditioning. Annu Rev Neurosci 11: 329–352. 10.1146/annurev.ne.11.030188.001553 3284445

[LM053726SENC67] Rescorla RA. 1988b. Pavlovian conditioning: it's not what you think it is. Am Psychol 43: 151–160. 10.1037/0003-066X.43.3.151 3364852

[LM053726SENC68] Rescorla RA, Wagner AR. 1972. A theory of Pavlovian conditioning: variations in the effectiveness of reinforcement and non-reinforcement. In Classical conditioning II: current research and theory (ed. Black AH, Prokasy WF), Vol. 2, pp. 64–69. Appleton-Century-Crofts, New York.

[LM053726SENC69] Rohwedder A, Wenz NL, Stehle B, Huser A, Yamagata N, Zlatic M, Truman JW, Tanimoto H, Saumweber T, Gerber B, 2016. Four individually identified paired dopamine neurons signal reward in larval *Drosophila*. Curr Biol 26: 661–669. 10.1016/j.cub.2016.01.012 26877086

[LM053726SENC70] Rolls ET. 2013. The mechanisms for pattern completion and pattern separation in the hippocampus. Front Syst Neurosci 7: 74. 10.3389/fnsys.2013.00074 24198767 PMC3812781

[LM053726SENC71] Rudy JW, Sutherland RJ. 1995. Configural association theory and the hippocampal formation: an appraisal and reconfiguration. Hippocampus 5: 375–389. 10.1002/hipo.450050502 8773252

[LM053726SENC72] Rybak J. 1994. Die strukturelle Organisation der Pilzkörper und synaptische Konnektivität protocerebraler Interneuronen im Gehirn der Honigbiene, *Apis mellifera*: eine licht-und elektronenmikroskopische Studie. Dissertation, FU Berlin, Berlin, Germany.

[LM053726SENC73] Rybak J. 2011. The digital honey bee brain atlas. In Honeybee neurobiology and behavior: a tribute to Randolf Menzel. pp. 125–140. Springer, Dordrecht, Netherlands.

[LM053726SENC74] Rybak J, Menzel R. 1993. Anatomy of the mushroom bodies in the honey bee brain: the neuronal connections of the alpha-lobe. J Comp Neurol 334: 444–465. 10.1002/cne.903340309 8376627

[LM053726SENC75] Sakagiannis P, Jürgensen A-M, Nawrot MP. 2021. A realistic locomotory model of *Drosophila* larva for behavioral simulations. bioRxiv 10.1101/2021.07.07.451470

[LM053726SENC76] Saumweber T, Husse J, Gerber B. 2011. Innate attractiveness and associative learnability of odors can be dissociated in larval *Drosophila*. Chem Senses 36: 223–235. 10.1093/chemse/bjq128 21227902 PMC3038274

[LM053726SENC77] Saumweber T, Rohwedder A, Schleyer M, Eichler K, Chen YC, Aso Y, Cardona A, Eschbach C, Kobler O, Voigt A, 2018. Functional architecture of reward learning in mushroom body extrinsic neurons of larval *Drosophila*. Nat Commun 9: 1104. 10.1038/s41467-018-03130-1 29549237 PMC5856778

[LM053726SENC78] Savastano HI, Miller RR. 1998. Time as content in Pavlovian conditioning. Behav Processes 44: 147–162. 10.1016/S0376-6357(98)00046-1 24896972

[LM053726SENC79] Schäfer S, Rehder V. 1989. Dopamine-like immunoreactivity in the brain and suboesophageal ganglion of the honeybee. J Comp Neurol 280: 43–58. 10.1002/cne.902800105 2918095

[LM053726SENC80] Scherer S, Stocker RF, Gerber B. 2003. Olfactory learning in individually assayed *Drosophila* larvae. Learn Mem 10: 217–225. 10.1101/lm.57903 12773586 PMC202312

[LM053726SENC81] Schlegel P, Bates AS, Stürner T, Jagannathan SR, Drummond N, Hsu J, Capdevila LS, Javier A, Marin EC, Barth-Maron A, 2021. Information flow, cell types and stereotypy in a full olfactory connectome. eLife 10: e66018. 10.7554/eLife.66018 34032214 PMC8298098

[LM053726SENC82] Schleyer M, Saumweber T, Nahrendorf W, Fischer B, von Alpen D, Pauls D, Thum A, Gerber B. 2011. A behavior-based circuit model of how outcome expectations organize learned behavior in larval *Drosophila*. Learn Mem 18: 639–653. 10.1101/lm.2163411 21946956

[LM053726SENC83] Schleyer M, Miura D, Tanimura T, Gerber B. 2015a. Learning the specific quality of taste reinforcement in larval *Drosophila*. eLife 4: e04711. 10.7554/eLife.04711 25622533 PMC4302267

[LM053726SENC84] Schleyer M, Reid SF, Pamir E, Saumweber T, Paisios E, Davies A, Gerber B, Louis M. 2015b. The impact of odor–reward memory on chemotaxis in larval *Drosophila*. Learn Mem 22: 267–277. 10.1101/lm.037978.114 PMC4408773 25887280

[LM053726SENC85] Schleyer M, Fendt M, Schuller S, Gerber B. 2018. Associative learning of stimuli paired and unpaired with reinforcement: evaluating evidence from maggots, flies, bees, and rats. Front Psychol 9: 1494. 10.3389/fpsyg.2018.01494 30197613 PMC6117914

[LM053726SENC86] Schleyer M, Weiglein A, Thoener J, Strauch M, Hartenstein V, Weigelt MK, Schuller S, Saumweber T, Eichler K, Rohwedder A, 2020. Identification of dopaminergic neurons that can both establish associative memory and acutely terminate its behavioral expression. J Neurosci 40: 5990–6006. 10.1523/JNEUROSCI.0290-20.2020 32586949 PMC7392503

[LM053726SENC87] Schroll C, Riemensperger T, Bucher D, Ehmer J, Völler T, Erbguth K, Gerber B, Hendel T, Nagel G, Buchner E, 2006. Light-induced activation of distinct modulatory neurons triggers appetitive or aversive learning in *Drosophila* larvae. Curr Biol 16: 1741–1747. 10.1016/j.cub.2006.07.023 16950113

[LM053726SENC88] Schröter U, Menzel R. 2003. A new ascending sensory tract to the calyces of the honeybee mushroom body, the subesophageal-calycal tract. J Comp Neurol 465: 168–178. 10.1002/cne.10843 12949779

[LM053726SENC89] Schröter U, Malun D, Menzel R. 2007. Innervation pattern of suboesophageal ventral unpaired median neurones in the honeybee brain. Cell Tissue Res 327: 647–667. 10.1007/s00441-006-0197-1 17093927

[LM053726SENC90] Stevenson PA, Spörhase-Eichmann U. 1995. Localization of octopaminergic neurones in insects. Comp Biochem Physiol A Physiol 110: 203–215. 10.1016/0300-9629(94)00152-J 7712064

[LM053726SENC91] Tabone CJ, De Belle JS. 2011. Second-order conditioning in *Drosophila*. Learn Mem 18: 250–253. 10.1101/lm.2035411 21441302 PMC3072777

[LM053726SENC92] Takeda K. 1961. Classical conditioned response in the honey bee. J Insect Physiol 6: 168–179. 10.1016/0022-1910(61)90060-9

[LM053726SENC93] Takemura SY, Aso Y, Hige T, Wong A, Lu Z, Xu CS, Rivlin PK, Hess H, Zhao T, Parag T, 2017. A connectome of a learning and memory center in the adult *Drosophila* brain. eLife 6: e26975. 10.7554/eLife.26975 28718765 PMC5550281

[LM053726SENC94] Thum AS, Gerber B. 2019. Connectomics and function of a memory network: the mushroom body of larval *Drosophila*. Curr Opin Neurobiol 54: 146–154. 10.1016/j.conb.2018.10.007 30368037

[LM053726SENC95] Tomasiunaite U, Widmann A, Thum AS. 2018. Maggot instructor: semi-automated analysis of learning and memory in *Drosophila* larvae. Front Psychol 9: 1010. 10.3389/fpsyg.2018.01010 29973900 PMC6019503

[LM053726SENC96] Tully T, Quinn WG. 1985. Classical conditioning and retention in normal and mutant *Drosophila melanogaster*. J Comp Physiol A 157: 263–277. 10.1007/BF01350033 3939242

[LM053726SENC97] Vosshall LB, Stocker RF. 2007. Molecular architecture of smell and taste in *Drosophila*. Annu Rev Neurosci 30: 505–533. 10.1146/annurev.neuro.30.051606.094306 17506643

[LM053726SENC98] Weiglein A, Gerstner F, Mancini N, Schleyer M, Gerber B. 2019. One-trial learning in larval *Drosophila*. Learn Mem 26: 109–120. 10.1101/lm.049106.118 30898973 PMC6432171

[LM053726SENC99] Widmann A, Artinger M, Biesinger L, Boepple K, Peters C, Schlechter J, Selcho M, Thum AS. 2016. Genetic dissection of aversive associative olfactory learning and memory in *Drosophila* larvae. PLoS Genet 12: e1006378. 10.1371/journal.pgen.1006378 27768692 PMC5074598

[LM053726SENC100] Winding M, Pedigo BD, Barnes CL, Patsolic HG, Park Y, Kazimiers T, Fushiki A, Andrade IV, Khandelwal A, Valdes-Aleman J, 2023. The connectome of an insect brain. Science 379: eadd9330. 10.1126/science.add9330 36893230 PMC7614541

[LM053726SENC101] Witthöft W. 1967. Absolute Anzahl und Verteilung der Zellen im Hirn der Honigbiene. Zoomorphology 61: 160–184.

[LM053726SENC102] Yamada D, Bushey D, Feng L, Hibbard K, Sammons M, Funke J, Litwin-Kumar A, Hige T, Aso Y. 2023. Hierarchical architecture of dopaminergic circuits enables second-order conditioning in *Drosophila*. eLife 12: e79042. 10.7554/eLife.79042 36692262 PMC9937650

[LM053726SENC103] Zars T, Fischer M, Schulz R, Heisenberg M. 2000. Localization of a short-term memory in *Drosophila*. Science 288: 672–675. 10.1126/science.288.5466.672 10784450

[LM053726SENC104] Zheng Z, Lauritzen JS, Perlman E, Robinson CG, Nichols M, Milkie D, Torrens O, Price J, Fisher CB, Sharifi N, 2018. A complete electron microscopy volume of the brain of adult *Drosophila melanogaster*. Cell 174: 730–743. 10.1016/j.cell.2018.06.019 30033368 PMC6063995

[LM053726SENC105] Zwaka H, Bartels R, Grünewald B, Menzel R. 2018. Neural organization of A3 mushroom body extrinsic neurons in the honeybee brain. Front Neuroanat 12: 57. 10.3389/fnana.2018.00057 30127725 PMC6089341

