# Supplementary material for: Cognitive limits of larval Drosophila: testing for conditioned inhibition, sensory preconditioning, and second-order conditioning
Source: Learn Mem. 2024 May;31(5):a053726. doi: 10.1101/lm.053726.122 (PMC11199949; doi:10.1101/lm.053726.122)
Supplement: Supplement 2 [file Supplemental_Figures.docx]

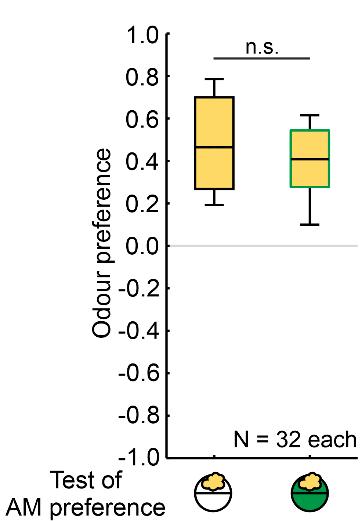


**Figure S1.** **Naïve odour preference is unchanged in the presence of a sugar reward**. Larvae were placed on a Petri dish filled with agarose (open circle) or sugar-supplemented agarose (green circle) together with one odour cup loaded with the indicated odour (yellow cloud) on one side and an empty odour cup on the other side of the Petri dish. After 3 min, the number of larvae on each side and in a middle zone were counted, and an odour preference was calculated according to Equation 1. The larvae showed approach to the odour regardless of whether the reward was present or not. Box plots represent the median as the midline, the 25/75% quantiles as box boundaries and 10/90% quantiles as whiskers. Sample sizes are indicated within the figure. n.s indicates non-significance in an MWU-test. Statistical results and source data in Supplemental Table S1.


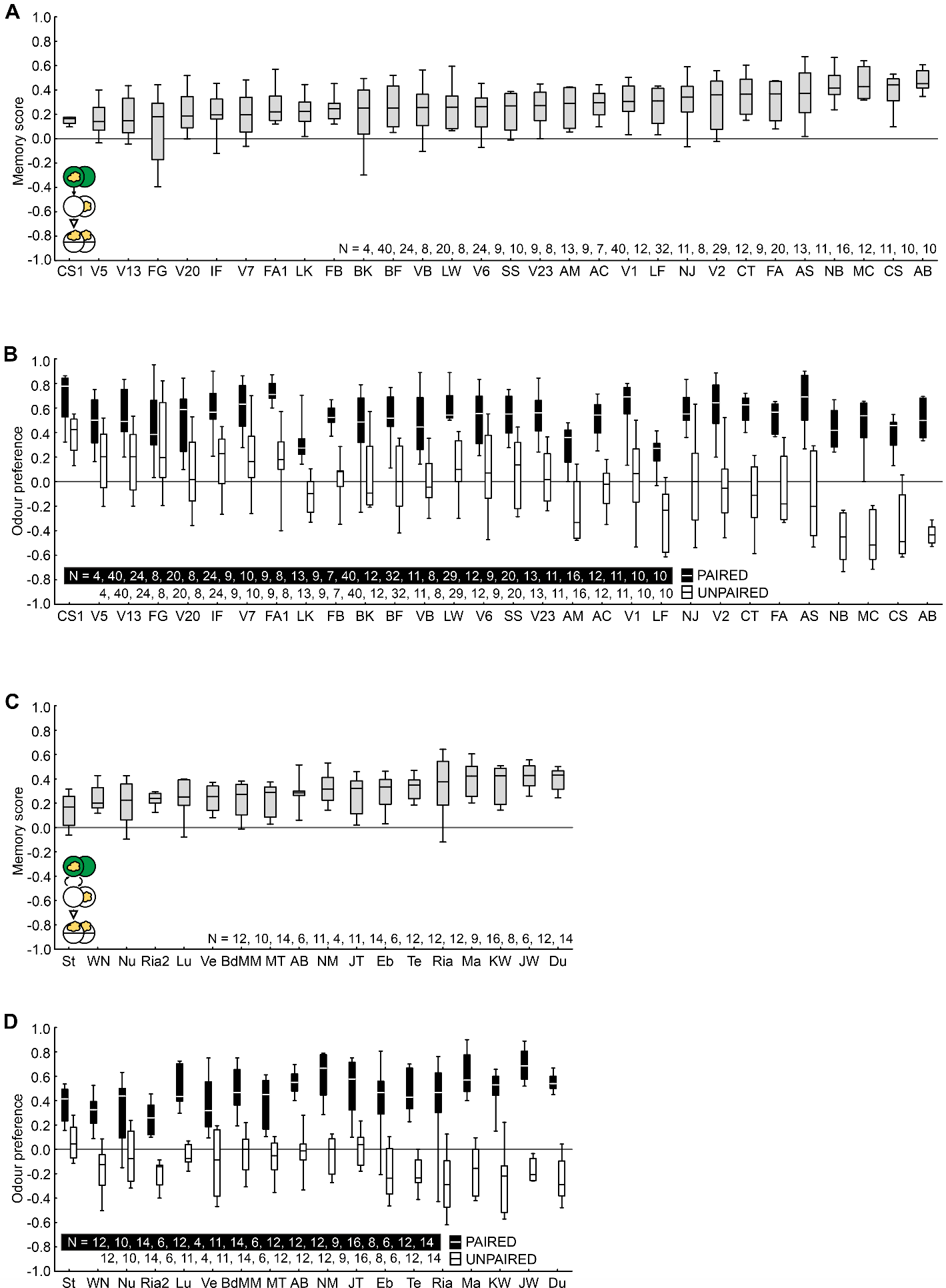


**Figure S2. Separation of the pooled data depicted in Fig. 4.** **(A)** Memory scores for the individual datasets underlying Fig. 4B, sorted by increasing median memory scores. **(B)** Odour preferences for the PAIRED (black boxes) and UNPAIRED (white boxes) trained groups underlying the memory scores in (A). **(C)** Memory scores for the individual datasets underlying Fig. 4F, sorted by increasing median memory scores. **(D)** Odour preferences for the PAIRED (black boxes) and UNPAIRED (white boxes) trained groups underlying the memory scores in (C). Letters below the plots refer to experimenters. Box plots represent the median as the midline, the 25/75% quantiles as box boundaries and 10/90% quantiles as whiskers. Sample sizes are indicated within the figure. Source data are given in the data file Supplemental Table S1.


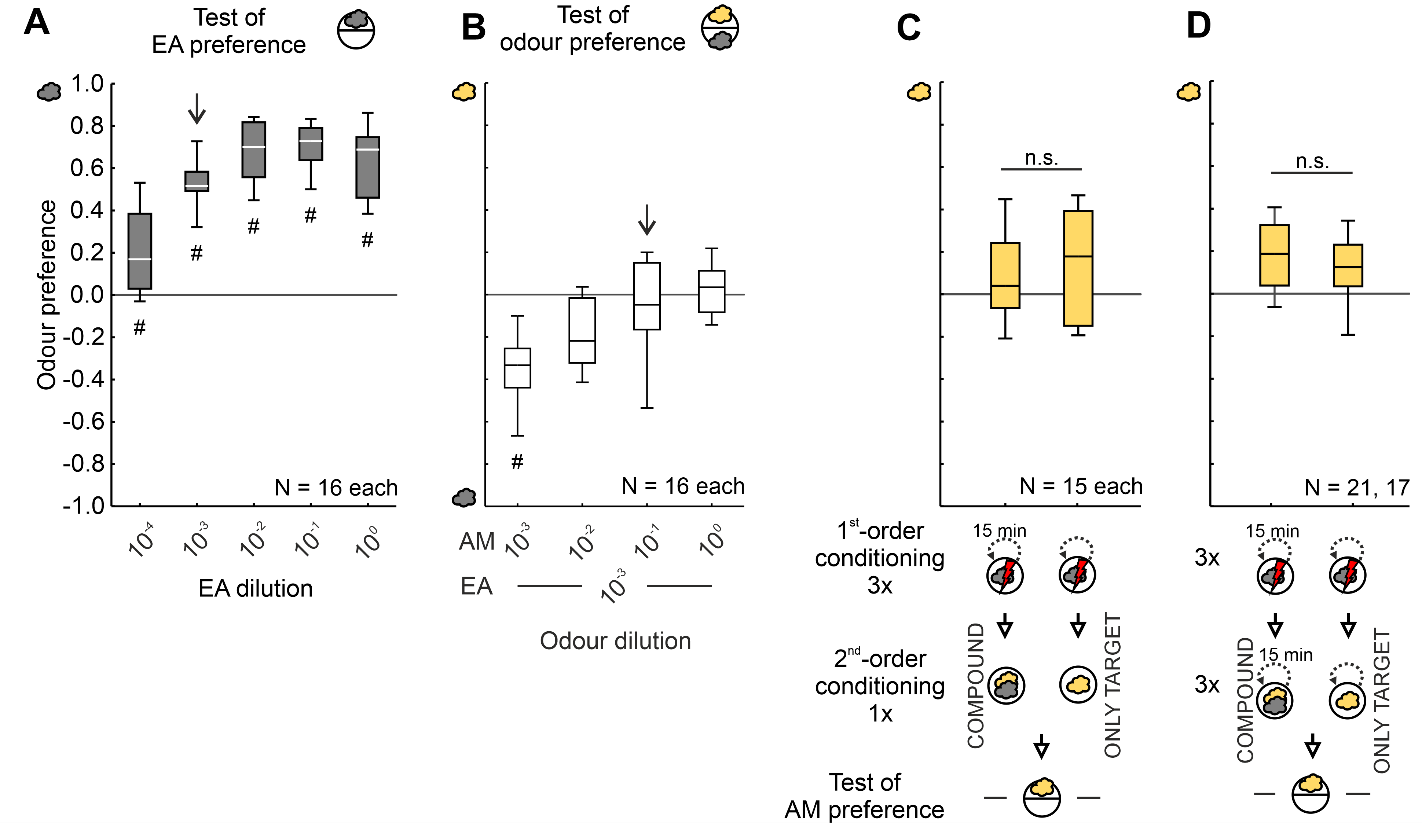


**Figure S3. No evidence of second-order conditioning in the aversive domain**. **(A-B)** Ethyl acetate (EA) (Sigma-Aldrich, Cat. No. 270989; CAS No. 141-78-6, diluted 10^-3^ in paraffin oil, CAS: 8042-47-5, AppliChem, Darmstadt, Germany) and amyl acetate (AM) (Sigma-Aldrich, Cat. No. 46022; CAS No. 628-63-7, diluted 10^-1^ in paraffin oil) were used as the trained and target odour, respectively. These dilutions were determined empirically to support moderate innate attraction to EA **(A)** (arrow) and a balanced choice between EA at such a moderately attractive dilution versus AM **(B)** (arrow). Paraffin oil is without behavioural significance to the larvae (Saumweber et al. 2011). Odour-electric shock associative learning experiments have previously been described in detail (Tomasiunaite et al. 2018). In brief, a custom-built semi-automatic device called "MaggotShock V 2.1" was used, which can transmit electric shock pulses through a custom-made setup with an 85-mm-diameter Petri dish filled with agarose (Sigma-Aldrich, Cat. No. A9539, CAS No. 9012-36-6). An electric shock stimulus from a DC power supply set to a total output of 80 V consisted of 30 pulses, each lasting 250 ms and followed by 250-ms breaks, for a total of 15 s. During conditioning, odours were presented on filter papers centred on the Petri dish lid. For the test, the larvae were transferred to the centre of a fresh 85-mm-diameter Petri dish equipped with a custom-made Teflon container with perforated lids and loaded with the target odour; these odour containers were randomly placed on the left or right side of the Petri dish. After 2 min the position of the larvae was noted, and a preference score for the target odour was calculated according to Equation 1. **(C-D)** During first-order conditioning, the larvae were placed onto the Petri dish, where they received the trained odour, followed 10 s later by electric shock for another 15 s. Coinciding with the offset of electric shock, the odour was removed, and the larvae were left undisturbed for 15 min. This cycle was repeated two more times. During second-order conditioning, the COMPOUND groups received the trained and the target odour in compound for 25 s, either only once (**C**) or three times (**D**, at 15-min intervals), whereas the ONLY TARGET group received the target odour only. For both groups, a test for the preference for the target odour followed. Lower odour preferences in the COMPOUND than in the ONLY TARGET group would offer preliminary evidence for second-order conditioning. No such difference was observed. Box plots represent the median as the midline, the 25/75% quantiles as box boundaries and 10/90% quantiles as whiskers. Sample sizes are indicated within the figure. # indicates significance from zero in an OSS test, n.s. indicates non-significance in an MWU-test. Source data are given in the data file Supplemental Table S1.
